# Cross-species neuroscience: closing the explanatory gap

**DOI:** 10.1098/rstb.2019.0633

**Published:** 2020-11-16

**Authors:** Helen C. Barron, Rogier B. Mars, David Dupret, Jason P. Lerch, Cassandra Sampaio-Baptista

**Affiliations:** 1 Medical Research Council Brain Network Dynamics Unit, Nuffield Department of Clinical Neurosciences, University of Oxford, Mansfield Road, Oxford OX1 3TH, UK; 2 Wellcome Centre for Integrative Neuroimaging, University of Oxford, FMRIB, John Radcliffe Hospital, Oxford OX3 9DU, UK; 3 Donders Institute for Brain, Cognition and Behavior, Radboud University, 6525 AJ Nijmegen, The Netherlands; 4 Department of Medical Biophysics, University of Toronto, Toronto, Ontario, Canada M5G 1L7; 5 Institute of Neuroscience and Psychology, University of Glasgow, Glasgow G12 8QB, UK

**Keywords:** cross-species, integrative neuroscience, animal model, non-invasive, microcircuit, behaviour

## Abstract

Neuroscience has seen substantial development in non-invasive methods available for investigating the living human brain. However, these tools are limited to coarse macroscopic measures of neural activity that aggregate the diverse responses of thousands of cells. To access neural activity at the cellular and circuit level, researchers instead rely on invasive recordings in animals. Recent advances in invasive methods now permit large-scale recording and circuit-level manipulations with exquisite spatio-temporal precision. Yet, there has been limited progress in relating these microcircuit measures to complex cognition and behaviour observed in humans. Contemporary neuroscience thus faces an explanatory gap between macroscopic descriptions of the human brain and microscopic descriptions in animal models. To close the explanatory gap, we propose adopting a cross-species approach. Despite dramatic differences in the size of mammalian brains, this approach is broadly justified by preserved homology. Here, we outline a three-armed approach for effective cross-species investigation that highlights the need to translate different measures of neural activity into a common space. We discuss how a cross-species approach has the potential to transform basic neuroscience while also benefiting neuropsychiatric drug development where clinical translation has, to date, seen minimal success.

This article is part of the theme issue ‘Key relationships between non-invasive functional neuroimaging and the underlying neuronal activity’.

## 1. Introduction: the explanatory gap

Measuring neural activity in the brain and relating it to complex behaviour remains a central challenge for contemporary neuroscience. In humans, this venture is limited by the non-invasive tools and techniques currently available. Magnetic resonance imaging (MRI) and magnetoencephalography (MEG), for example, are restricted to coarse measures of neural activity that aggregate the diverse responses of thousands of neurons over space and time. These tools provide macroscopic measures of cognitive processing that relate to human behaviour but fail to provide insight into neural activity at the microcircuit, cellular and synaptic levels. To investigate neural activity at the microscopic level, to reveal the division of labour across cell types in their host circuit and assess causality, we instead rely on invasive procedures in animal models. In recent years, we have seen the development of new recording techniques that can simultaneously monitor activity from thousands of cells across numerous brain regions. Furthermore, the expansion in the use of genetic tools in rodents now permits manipulation of neural activity at unprecedented spatio-temporal resolution. Yet, in contrast with research carried out in humans, these approaches rarely characterize activity at the macroscopic level and interpreting animal behaviour is challenging. This makes it difficult to establish how neural mechanisms recorded and manipulated in animal models relate to higher-order cognition.

Owing to the distinct training requirements for neuroscientists conducting research in humans or animal models, laboratories typically employ a species-specific approach where research is focused on only one species. By and large, this centres research on either the macroscopic or microscopic level, leaving an explanatory gap between genetic, (sub)cellular and circuit-level mechanisms on the one hand, and higher-order cognition on the other. The adverse implications of this explanatory gap are made evident by high failure rates observed in clinical trials, where neuropsychiatric drugs have one of the highest failure rates at Phase III [1]. With an ageing global population, neuropsychiatric disease presents an increasing social and economic burden that the World Health Organization (WHO) describe as the major public health problem of all high-income countries [2]. There is, therefore, an urgent need to develop a more integrated approach to neuroscientific research, one that seeks to close the explanatory gap between human and animal research.

Here, we explore the view that investing in an interdisciplinary, cross-species approach will provide a means to integrate different levels of neuroscientific description, paving the way for a comprehensive understanding of how the mammalian brain serves adaptive behaviour. We outline a three-armed approach for effective cross-species investigation. First, to provide appropriate interpretation of non-invasive methods, different tools (i.e. both non-invasive and invasive methods) need to be employed within the same species. Second, to provide a direct means to relate signals recorded across different species, the same tools need to be employed across multiple species. Third, to obtain complementary datasets that take advantage of the best tools available in each species, different tools should be employed across different species using a comparative approach. Thus, by complementing current approaches that provide detailed descriptions of neural processing within one species, or even within one brain region, a cross-species approach may uncover a set of general principles that describe the neural basis of cognition and behaviour in terms of cellular and circuit-level mechanisms. Moreover, adopting a cross-species approach may harness the translational value of fundamental neuroscience to develop effective neuropsychiatric treatment.

## 2. What can we measure in humans?

Each tool used for measuring neural activity has its own advantages and limitations. Of the non-invasive techniques available for measuring brain activity, electroencephalogram (EEG), MEG and functional MRI (fMRI) all provide readouts of activity at the macroscopic level.

The temporal resolution of EEG and MEG out-performs that of fMRI, and while EEG has poor spatial resolution, MEG can match the spatial resolution of fMRI in cortical brain regions. MEG uses highly sensitive magnetometers to measure the weak magnetic fields generated by electrical activity of neuronal populations within the brain [3]. The recorded signal is thought to reflect fluctuations in membrane potential across many neurons, with the amplitude depending upon the number of active neurons, their temporal synchrony and spatial alignment [4]. The temporal resolution (on the order of milliseconds) is sufficiently high to probe oscillatory neuronal dynamics that directly map to the local field potential measured using invasive electrophysiology in animal models. Moreover, the evoked potential can be used to study language and auditory processing [5,6], while rapid changes in the spectral amplitude of oscillations over time can be used to decode neuronal representations during working memory maintenance [7] and memory recall [8].

Conventional MEG uses superconducting quantum interference devices (SQUID). However, these sensors require cryogenic cooling together with thermal insulation, which limits the proximity between the SQUID and the subject's scalp. Recent developments have introduced new scalp-mounted devices that operate at room temperature using optically pumped magnetometers (OPMs) [9–12]. These new sensors offer a significant advantage over SQUIDs as they can be placed directly on the scalp, increasing the magnitude of the measured signal [13] but also permitting signal acquisition as the participant moves [10].

fMRI, on the other hand, is more widely available than MEG and provides a means to image the entire brain at relatively high spatial resolution. fMRI has the advantage of being readily compared with other imaging modalities that provide insight into brain anatomy, connectivity and chemical composition, or combined with causal interventions such as non-invasive brain stimulation. However, its interpretation is not straightforward: the blood oxygen level dependent (BOLD) signal measured using fMRI provides only an indirect measure of neural activity and the relationship between neural activity and the BOLD signal is complex [14,15]. Remarkably, despite multiple opportunities for nonlinearity (for example, the relationship from stimulus to neural activity; and the relationship between neural activity and the BOLD signal), evidence suggests the relationship between neural firing rate and the BOLD signal is approximately linear, at least over a limited range [16–22]. This approximately linear relationship underpins the use of fMRI as an effective tool to infer neural activity using a non-invasive method.

While fMRI boasts the highest spatial resolution of available non-invasive methods, even submillimetre ultra-high-field fMRI includes tens of thousands of neurons per voxel. Researchers have, therefore, developed methodological approaches to map the coarse spatial organization of neurons. For example, fMRI can be used to measure retinotopic [23–25], tonotopic [26,27] and somatotopic [25,28] maps that resemble topographic maps measured using invasive methods in animal models. Topographies that span connections (connectopies) may also be used to decipher the overarching principles of organization inherent to different brain regions in different individuals [29,30]. Moreover, these methodological approaches have clinical relevance, where somatotopic mapping in the primary somatosensory cortex can be used to measure the persistent digit topography of amputees' missing hand [31], while retinotopic mapping in V1 can be used to characterize the relative plasticity and stability of visual cortex in patients with congenital visual pathway disorders [32,33].

The improved spatial resolution afforded by an increase in signal-to-noise ratio at high-field strength has further opened up the possibility for columnar fMRI [34,35] and layer-specific (laminar) fMRI [36–39]. In contrast with traditional fMRI, which captures the amalgamation of both feed-forward and feedback responses [40], submillimetre resolution fMRI can begin to dissociate the functional role of feed-forward and feedback projections that activate different cell layers within the cortex. For example, in human V1, consistent with the known anatomy [41,42], laminar fMRI shows that responses attributed to top-down feedback selectively activate deep cortical layers, such as the representation of an occluded part of an object or an illusory shape [43,44]. However, despite providing a unique opportunity to measure cortical organization *in vivo* at a resolution previously restricted to invasive methods in animals [45–50], laminar fMRI is affected by sequence-dependent and depth-dependent draining artefacts attributed to uneven vascular architecture [39,51]. Reliable deployment of high-field fMRI, therefore, requires a detailed understanding of neurovascular coupling.

Alongside improvements in spatial resolution, recent advances in fast scanning techniques have pushed the temporal resolution of fMRI. These include multiband and simultaneous multi-slice sequences that achieve subsecond sampling [52–54]. The temporal resolution of fMRI, however, remains fundamentally limited by the slow nature of the haemodynamic response function (HRF), which peaks at approximately 5 s after stimulation, and is followed by an undershoot that lasts approximately 30 s [55]. Overlap between successive events can be explicitly modelled under the assumption that the responses add in a linear fashion [56,57]. However, when the inter-stimulus interval is below about 1.5 s, ‘saturation’ in the mapping from neural activity to the BOLD signal introduces nonlinearities [58,59] that cannot easily be accounted for using the standard analysis pipelines.

To measure neural events at a subsecond resolution requires alternative analytical approaches. Recent fMRI investigations demonstrate that relatively rapid neural sequences (on the order of a few hundred milliseconds) may be decoded using multivariate decoding techniques that assess subtle differences in the activity patterns across voxels, measured across consecutive repetition times (TRs) [60]. Simulations further suggest this approach is, in principle, sensitive to sequential neural events that occur on the order of 100 ms [60]. The ability to decode these relatively rapid neural sequences using fMRI can be understood as the consequence of temporal blurring of neural events by the HRF. Two neural events within the same multi-step sequence will affect the BOLD signal over several seconds, thus being represented by consecutive TRs. During periods of rest or sleep, this approach, along with recent developments using MEG [7,8,61,62], may be used to measure sequential activity patterns in the hippocampus, analogous to ‘replay’ spiking activity previously reported using invasive hippocampal electrophysiological recording in rodents [63–65]. Hippocampal ‘replay’ involves accelerated reactivation of specific spiking activity patterns previously observed during the wake/active state and is thought to play a key role in memory consolidation and planning [66–68]. Using non-invasive, whole-brain methods to measure relatively rapid activity patterns in humans may provide insight into how hippocampal ‘replay’ influences higher-order cognition and activity in other brain regions [60,62].

But despite these improvements in spatio-temporal resolution and analytical approaches, fMRI and other non-invasive methods, such as MEG, continue to provide only limited insight into cellular and synaptic processes that characterize neural activity at the microcircuit level. Therefore, while ongoing research is continuing to deepen our understanding of the relationship between specific neuronal subtypes and different vascular variables that affect the BOLD signal [69–71], certain neurophysiological processes simply cannot be measured non-invasively. Even with a dramatic advance in the spatio-temporal resolution of non-invasive methods, *in vivo* non-invasive recordings of the human brain will at best provide an *index* or *indirect measure* for activity at the subvoxel resolution, as demonstrated by innovative approaches showing insight into neural codes [72], temporal sequences [60–62], synaptic plasticity [31,73,74] and excitatory and inhibitory processes [74–76]. The validity of these measures, the discovery of new principles of microcircuit organisation and the precise contribution made by different cell types to neural computation will continue to rely on invasive recordings in animal models.

## 3. What can we measure in animal models?

Except in unusual circumstances, such as during electrocorticographic and depth recordings in epilepsy and deep brain stimulation patients [77,78], ethical restrictions limit the study of the human brain to non-invasive methods. Although this may change in the near future, with the advent of implantable bidirectional devices that piggy-back chronic neurophysiological recording capabilities on the delivery of chronic therapeutic stimulation, such opportunities will remain confined to selected conditions or disease states. To monitor and manipulate physiological neural activity at the cellular, synaptic and circuit level, we instead rely on invasive methods in animal models. Recent technological developments in invasive methods now permit large-scale and long-term recording in animal models, alongside manipulation of neural activity at unprecedented spatio-temporal resolution.

Invasive methods available for recording neural activity during behaviour include *in vivo* electrophysiology that has temporal resolution sufficient to resolve individual action potentials, the fundamental currency of neural information. The micro-machined silicon probes developed in recent years, such as neuropixels [79], can be used to simultaneously record activity from thousands of neurons across numerous brain regions [80], thus representing an important advance from traditional recording techniques. The introduction of polymer electrode-based systems further supports stable single-unit recording with longevity extending to five months or more [81]. When coupled with automated spike sorting methods [82,83] and sophisticated analysis pipelines, large-scale electrophysiology can begin to reveal the organizing principles, distribution and character of neural activity supporting behaviourally relevant variables [84]. Furthermore, the relationship between neuronal spiking and the local field potential can be used to reveal how synchronized networks and particular oscillatory patterns support effective neuronal communication during well-defined behaviours [85,86].

While distinct cell types, including excitatory and inhibitory neurons, may be deduced from electrophysiological features, complementary methods must typically be employed to cross-validate identified neuronal types [87–91]. Notably, recent advances in genetic tools afford the necessary specificity and precision to relate the function of particular neuronal subtypes to well-defined behaviour in rodents [92]. When combined with highly sensitive optical probes used for imaging intracellular calcium (a proxy for spiking activity) [93], genetic tools can also be employed to dissociate distinct interneuron subtypes within neural circuits [94,95]. In the worm [96], zebrafish [96–98] and *Drosophila* [99], genetically encoded calcium indicators permit whole-brain imaging, a powerful approach for establishing the relationship between brain-wide circuits and behaviour. However, interpreting neuronal calcium signalling is not straightforward [93]. While spiking activity in neurons triggers large changes in the concentration of cytoplasmic-free calcium, the resulting intracellular calcium dynamics are slow and derive from multiple sources that sum nonlinearly. Despite iterative improvement in the sensitivity and kinetics of calcium indicators, it remains highly challenging to deconvolve single action potentials from calcium transients. Instead, the ongoing development of membrane voltage indicators promises a tool that provides both genetic targeting and temporal precision with subthreshold sensitivity [100].

Particularly in small animals, genetic tools further support causal manipulations, such as optogenetics where light is used to control neural activity with cell-type and millisecond precision [101,102]. The specificity and breadth of optogenetic methods support both activation and inactivation experiments. When combined with well-defined behavioural tasks, these methods provide a toolkit to relate physiological mechanisms to behaviour.

These readouts and manipulations of microcircuit-level activity go hand-in-hand with an understanding of structural neuroanatomy where axonal tracing in animal models still provides what is often termed the ‘gold-standard’. Such invasive tools are currently the only methods available for identifying the direction of a connection and the presence of synapses. While non-invasive anatomical methods, such as diffusion-weighted MRI-based tractography, have the advantage of providing *in vivo* reconstructions and visualization of the three-dimensional architecture of white matter tracts, they do not trace axons directly, and variables such as crossing fibres and fibre geometry, among others, influence the accuracy of the results. Therefore, results need to be carefully interpreted and often validated in animal models when possible.

Invasive methods in animal models are, however, not without their own limitations. Hubel & Wiesel [103], who pioneered some of the earliest use of electrophysiology in the late 1950s, recognized the drawbacks of their approach: ‘to attack such a three-dimensional problem with a one-dimensional weapon is a dismaying exercise in tedium, like trying to cut the back lawn with a pair of nail scissors'. Despite recent developments, these criticisms do, in part, still ring true: electrophysiology can be biased towards the large spikes discharged by a subset of neurons, leading to under-sampling of smaller spikes discharged by other neuron types. Moreover, electrophysiology typically samples a subset of neurons at a restricted location, potentially overlooking the macroscopic structure of neural activity and system-wide dynamics. And even when large numbers of neurons are recorded simultaneously, interpreting the neural activity is no mean feat, somewhat analogous to trying to decipher the ‘operation and function of an orchestra, without knowing much about the role of strings, woodwinds, brass or percussion instruments’ [104].

The rapidly expanding use of optical and genetic tools available in rodents has also been met by growing recognition of the pros and cons associated with these methods. For instance, the slow kinetics of calcium imaging complicate interpretation of the signal [93], and voltage indicators currently have limited brightness and photostability to support *in vivo* imaging during ongoing behaviour. Optogenetic stimulation risks driving neuronal responses outside their typical physiological range, causing bulk activation and the potential for unnatural plasticity; and the resulting behavioural effects may reflect the function of a manipulated circuit, as opposed to a loss- or gain-of-function manipulation.

However, perhaps the most pressing concern is simply that these contemporary invasive tools are predominantly employed in rodents. Rodents are used as model organisms that allow comprehensive measurement and manipulation, but research is restricted to the repertoire of rodent behaviours that are easy to interpret. This may in part be overcome by improved characterization and quantification of ethological rodent behaviour using more precise and automated tools [105,106]. However, difficulties will persist in relating rodent behaviour to higher-level cognition observed in humans. While behaviours in non-human primates are arguably more closely aligned with those in the human, non-human primate research will always be limited by numbers. These limitations of invasive research in animal models have implications for fundamental and translational neuroscientific research. The stark consequence of these shortcomings is perhaps most evident in psychiatric research, where the full complexity of disorders can rarely, if ever, be modelled (see §10).

## 4. Can a cross-species approach bridge the macroscopic–microscopic divide?

Having examined current state-of-the-art tools available for investigating neural activity in both humans and animals, the explanatory gap between non-invasive and invasive tools is evident and highlights the limitation of a species-specific approach. On the one hand, non-invasive methods available in humans can relate measures of macroscopic activity to complex cognition and behaviour. Yet, these non-invasive techniques are limited by poor spatial or temporal resolution, and, at least for fMRI, they provide an indirect measure of neural activity. On the other hand, invasive methods available in animal models can measure neural activity and synaptic changes at high spatio-temporal resolution, but often limit investigation to a single neural circuit or brain region and behaviours that are easy to interpret. Microscopic measures in animal models therefore fall short of providing insight into distributed computations that underlie the diverse and complex repertoire of human behaviour.

Can we use a cross-species approach to bridge the macroscopic–microscopic divide? After all, different species have different lifestyles, occupy and adapt to different ecological niches, and are exposed to different evolutionary pressures. While these different evolutionary pressures may in part account for differences between species [107,108], overall we see that preserved structure and function of neural circuits and the encoded sequences within the human genome are highly overlapping with that of other mammals (99% overlap between human and mouse, for example) [109,110]. More substantial differences are observed in gene expression at the cellular level (79% overlap between humans and mice, for example), but species-specific expression differences appear to have discrete, non-widespread expression patterns that are considered to reflect subtle rather than global changes [111]. Thus, despite important differences, the general organization of neural circuits within the mammalian brain appears conserved.

At the structural level, early work by Brodmann [112] and others revealed the cytoarchitectural organization of cortex across species. Researchers have since shown that while some species-to-species variability in neuronal subtypes does exist [113–115], by and large, the same neuronal subtypes, defined by molecular expression profiles and dendritic patterns, can be found in the same brain regions of humans and other mammals [116,117]. For example, in both humans and rats, axo-axonic GABAergic cells show equivalent innervation patterns and initiate a stereotyped series of synaptic events in cortical networks [118]. The interaction between different neuronal subtypes together forms the basic microcircuit that appears to have been replicated several thousand times in larger mammalian brains [108]. Therefore, despite 17 000-fold variability in brain volume leading to substantial differences in the number of brain areas across the mammalian order [119–121], the general principles of organization, defined by neuronal subtypes and microcircuit structure, appear broadly conserved. Arguably, this means that even brain regions or neural circuits that are uniquely human may be understood using a set of general principles that derive from animal models [122].

**Figure 1.**
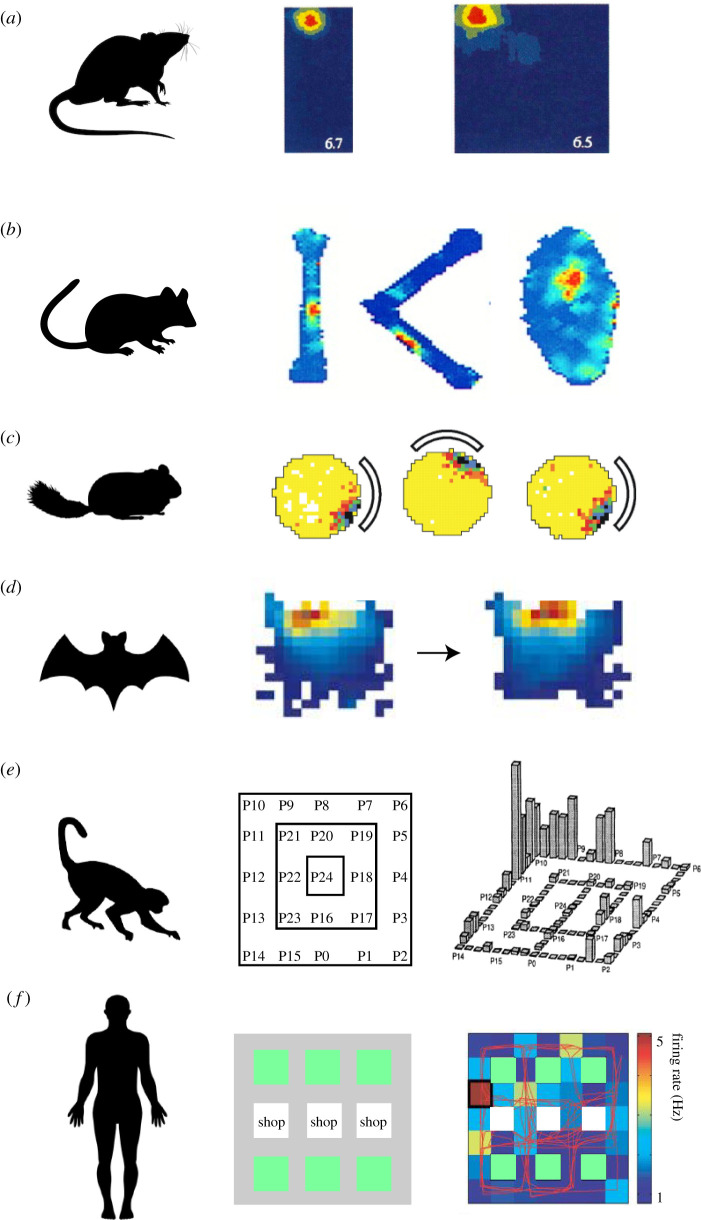
Place cells in the hippocampus of different mammalian species. Electrophysiology recordings in the hippocampus show evidence for ‘place cells’ across different mammals. As animals/humans traverse an environment, place cells show increased firing at a specific location in the environment, in: (*a*) rats [136]; (*b*) mice [132]; (*c*) chinchillas [133]; (*d*) bats [134]; (*e*) monkeys [135] and (*f*) in humans navigating a virtual environment [77].

Similarly at a functional level, resting-state fMRI in primates reveals a remarkably conserved profile for functional connectivity across large-scale networks such as the default mode network (macaque: [123]; chimpanzee: [124]), with similar connectivity hubs across species [125]. In both humans and non-human primates, similar functional responses have also been observed during visual processing [126], tool use [127], sequence processing [128] and decision-making [129]. Furthermore, in the hippocampus, a brain region situated towards the apex of the visual processing hierarchy [130], neurons show equivalent functional significance across mammals. Indeed, ‘place cells’—neurons that are activated when animals pass through a specific location in the environment have been identified in the hippocampus in rats [131], mice [132], chinchillas [133], bats [134], monkeys [135] and humans [77] (figure 1). In addition, in all tested species, place cells in the CA1 region of the hippocampus are reported to be pyramidal cells that have characteristic bursting activity with peak firing rates residing within a similar range [137]. The significance of these cross-species comparisons is that place cells are reported to constitute a cognitive map that aids high-level cognitive function, including navigation, planning and memory [138].

Thus, as we move to larger brains, compensatory mechanisms appear to preserve brain-size-invariant neural dynamics and computation. Signal delay caused by increasing transmission distance is offset by increasing axon size and myelination, which increase conduction velocity and reduce signal attenuation. A minority of disproportionately large axons further help preserve transmission time while minimizing the cost of increasing brain volume [139,140]. Across mammals, these compensatory mechanisms appear to preserve neural codes, temporal dynamics and the core function of neural circuits.

## 5. Developing a cross-species approach

**Figure 2.**
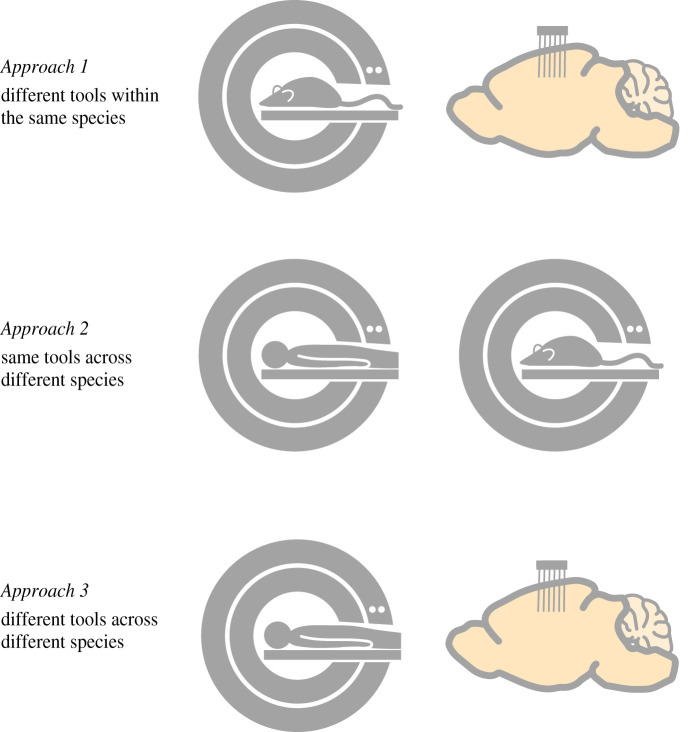
A three-armed approach for efficacious cross-species research. To bridge the explanatory gap between macro- and microcircuit measures of neural activity, we propose a three-armed cross-species approach. First, different tools need to be simultaneously employed within the same species to aid appropriate interpretation of non-invasive methods (*Approach 1*). Second, the same tools need to be employed across different species to perform comparative investigations (*Approach 2*). Third, different tools should be employed in parallel across different species, to provide state-of-the-art measures of neural activity at both a macro- and microcircuit level, while employing methods to translate neural signatures across different recording modalities (*Approach 3*).

Preserved homology of neural circuits across mammals underpins the rationale for conducting investigations across multiple species. But even when investigating aspects of cognition that are considered to have uniquely human components, such as language, a comparative cross-species approach (e.g. between humans and non-human primates) can reveal structural and functional specialization [141]. Thus, a cross-species approach may be used to bridge the gap between human neuroimaging and invasive animal research. Here, we outline three complementary approaches for efficacious cross-species investigation (figure 2).

### (a) Approach 1

Different tools need to be simultaneously employed within the same species to provide appropriate interpretation of non-invasive methods. With regard to fMRI, the relationship between the BOLD signal and neural activity can be characterized in animal models by simultaneous fMRI and electrophysiological recordings [142–144], or by optical imaging of both neural activity and haemodynamics [145]. By continuing to combine measures of the BOLD signal with invasive recording, *Approach 1* will establish a deeper understanding of the relationship between the BOLD signal and the underlying neural activity. Since the relative merit of this approach and interpretation of the BOLD signal have been detailed elsewhere [14,15,146], in this opinion piece, we will only consider *Approach 1* in passing.

### (b) Approach 2

The same tools need to be employed across multiple species, to allow direct comparisons to be drawn between different species. For example, to reveal functional properties that generalize across species, MRI may be used to perform comparative investigations (see §6). Alternatively, electrophysiology may be employed across different animal models and compared with pre-operative recordings in epilepsy patients. Functional comparisons can be established by matching behavioural assays (see §7).

### (c) Approach 3

The third approach takes advantage of behavioural assays that can be implemented across species but uses the best tools available in each species to characterize the macroscopic and microscopic levels in tandem. To compare complementary datasets, this approach requires quantitative analytical approaches that translate different measures of neural activity into a common space (see §§§7,8,9). In this manner, data obtained from different recording modalities can be directly compared. This third approach can thus facilitate an interplay between human and animal research that goes beyond the sum of its parts.

## 6. Cross-species magnetic resonance imaging

The same tools need to be employed across multiple species (*Approach 2*). Cross-species MRI seeks to do exactly this, using non-invasive MRI to quantify neural structure and function *in vivo* across both animals and humans. First, comparable signals can be obtained across species, providing a means to assess structural and functional homology while also identifying brain regions and connections unique to a particular species [147–150]. Second, cross-species MRI can be combined with invasive methods available in animal models. Therefore, histology, optogenetic manipulations and other invasive methods can be carried out after or in combination with MRI assessments. Although potential limitations must be acknowledged [151], cross-species MRI has the potential to bridge the divide between aggregate measures of neural activity acquired with imaging and microcircuit-level activity measured with invasive methods.

At a structural level, diffusion-weighted MRI-based tractography can be used to provide direct anatomical comparisons across species, with validation using tract-tracing techniques and histology. For example, direct structural comparisons can be made between human and macaque cortex using surface-based registration to align a few known homologous cortical landmarks. Evolutionary expansion maps generated using this approach can reveal areas in the human brain that have disproportionally expanded [152]. Alternatively, connectivity blueprints can be generated for each brain region (or grey matter vertex), and for each species. Within a common space, these connectivity profiles can then be compared to identify common principles and homologies between species, while also revealing unique specializations [153]. For example, when comparing the human brain with the macaque and chimpanzee brain, a large expansion can be observed in the arcuate fasciculus that mediates frontal–temporal connections, suggesting evolutionary divergence since our most recent common ancestor 6 million years ago [120,154,155]. Arguably, these comparative investigations reveal evolutionary relationships between species, while also delineating key differences that obviate the possibility for direct comparison [153].

Perhaps the real versatility of cross-species MRI becomes apparent when considering small-animal MRI. Small-animal MRI, in mice and rats, is complicated by the small size of the rodent brain (approx. 0.4 g in mouse versus approx. 1.4 kg in humans). Yet, recent developments in cryo-coils [156], optimized imaging sequences and ultra-high-field imaging ensure sufficient signal-to-noise for submillimetre spatial resolution. Small-animal MRI can, therefore, support reliable whole-brain fMRI in rodents and can be coupled with invasive methods that characterize neural circuits and establish causal specificity. Particularly in mice, this opens up an opportunity to take advantage of transgenic lines and genetically engineered mouse models that can be combined with multiple invasive methods. Small-animal MRI, therefore, provides a unique opportunity to characterize microcircuits while concomitantly acquiring whole-brain signatures of neural activity during behaviour.

Small-animal MRI is predominantly carried out in anaesthetized or sedated animals, primarily owing to the requirement to hold the head in the same position during imaging. This makes small-animal MRI highly suitable for studies investigating structural changes throughout development and ageing and in response to interventions [157]. Long-lasting structural changes attributed to learning can be observed via regional changes in brain volume [158,159], or diffusion properties [160–162], even after only 1 day of learning [163]. With the introduction of quantitative imaging and microstructural modelling approaches, structural imaging is moving closer to accurate estimates of neural morphometry [164–166].

Under anaesthesia, small-animal fMRI has been acquired during stimulus-evoked paradigms to successfully map layer-specific BOLD activation [167], whole-brain circuits [168] and monitor recovery and interventions following experimental stroke models [169]. However, given the technical challenge associated with these approaches, small-animal fMRI is more commonly used to probe whole-brain functional connectivity (resting-state fMRI) [170]. Although small-animal resting-state fMRI is subject to variability in preclinical equipment, animal handling protocols and sedation regimes, recent multi-centre comparisons show how standardized pre-processing pipelines and analytical steps can promote reproducibility and facilitate meta-analysis [151]. When these standardized pipelines are applied to multi-site mouse resting-state fMRI, spatially defined motifs and local connectivity show a high degree of convergence across datasets [171]. When implemented across species, resting-state fMRI provides a tool to reveal macroscopic organization common to the mammalian brain, by permitting comparison between functional connectivity fingerprints in rodents, non-human primates and humans [172].

Moving beyond tools that facilitate direct comparison between animals and humans, small-animal imaging has seen recent advances in multi-modal imaging where fMRI is combined with invasive neurophysiological measures (*Approach 1*). For example, multi-modal imaging can reveal linear fits between neuronal and capillary responses (two photon microscopy) and mesoscopic responses detected with BOLD fMRI, demonstrating that even low levels of neuronal activation can trigger elevations in blood flow [173]. Combined fMRI with optogenetically driven neuronal calcium signals can further be used to identify neurovascular coupling patterns at the level of a single vessel [174]. For example, optic fibre-based calcium recordings of neural populations local to cortex and thalamus can now be combined with whole-brain BOLD fMRI to relate slow-wave oscillations to the BOLD signal [175]. Together, these studies illustrate how multi-modal imaging can bridge the explanatory gap between different levels of neuroscientific inquiry and establish a more detailed understanding of the BOLD signal and its relationships with neurophysiology.

However, it is important to recognize the limitation of studies that rely on imaging animals under anaesthesia. Anaesthetics introduce known confounds to fMRI measurements. First, anaesthetized animals have lower baseline levels of neural spiking activity and show reduced BOLD signal intensity [176]. Second, anaesthetics affect cerebral blood flow and vasodilation, thus modulating the BOLD contrast itself [177]. Proper interpretation of the BOLD signal when using anaesthesia is further complicated by variability in vasodilation caused by different levels of anaesthesia, the use of different anaesthetics across studies [170] and different responses to anaesthesia across species. To separate the neural and vascular effects of anaesthesia on the BOLD signal, parallel acquisition of fMRI and calcium imaging can be implemented [81], highlighting how both the effect of anaesthetics and CO_2_ on the BOLD signal must be considered [178]. Despite these confounding effects, anaesthetic protocols are being identified that deliver long-lasting sedation with robust and time-invariant stimulus-evoked BOLD responses. For example, the administration protocol for the anaesthetic medetomidine has been clearly defined in rats, thus providing a suitable reference for protocols that require stable stimulus-evoked and resting-state fMRI in this species [179].

The alternative to using anaesthetics involves implementing imaging during awake behaviour but minimizing animal movement, and potential distress presents a significant challenge. Despite these technical difficulties, fMRI in awake behaving rodents has recently been demonstrated in a Pavlovian fear conditioning paradigm in rats [180] and in an inhibitory control task in mice [181].

The versatility of small-animal imaging has further led to widespread use of preclinical imaging as a test bed for pharmaceutical research. For example, preclinical imaging is now being used for high-throughput phenotyping of transgenic animals, profiling of new disease models, pharmacological and pharmacokinetic analysis for target identification, safety testing and evaluation of drug effects on host anatomy, function and metabolism [182,183]. The non-invasive nature of preclinical imaging renders longitudinal studies possible, along with experimental designs that use each animal as their own control. As most preclinical imaging techniques are analogous to those available in the clinical setting, results have the potential to be translated into humans [184,185]. Thus, this approach seeks to obtain non-invasive markers of neural activity that can be readily measured in human health and disease.

## 7. Cross-species behavioural assays

Although structural and functional homology across the mammalian brain broadly justifies adopting a cross-species approach, neural representations that support cognition cannot be measured and compared across species without comparable behavioural assays.

The systematic monitoring of overt behaviour in humans and animals began with the work of behaviourists in the early twentieth century. Work by Tolman [186], among others, further introduced the idea that overt behaviour may be considered the effect of a number of variables that include inputs from the environment (stimuli), but also motivational and emotional state, and internal representations of the environment stored within a ‘cognitive map’. This nuanced perspective of behaviour accounts for the rich and flexible repertoire observed in humans and animals, but also highlights the challenges associated with modelling human behaviour in animals. In the absence of direct communication, animal behaviour is difficult to interpret. Furthermore, some behaviours are difficult to model or simply considered unique to humans. The high failure rates reported in clinical trials for neuropsychiatric drugs may, in part, be attributed to poor behavioural assays that fail to either simulate or quantify the full complexity of behaviour observed in patients (see §10).

To take advantage of the potentially rich behavioural repertoire of animals, first we need to develop more advanced tools to quantify animal behaviour [105,106]. Second, we need to develop behavioural assays that can be implemented in both humans and animal models. One approach involves using virtual reality (VR) to simulate three-dimensional (3D) environments. VR provides a means to deliver sensory stimulation within a dynamic, immersive and realistic environment, while ensuring tight control over experimental variables during physiological and behavioural monitoring. By carefully considering species-specific differences in the processing and response to stimuli, including their perceived saliency, near-equivalent VR environments can be employed across multiple species [187]. In this manner, behavioural assays that employ VR can permit direct comparison of microscopic and macroscopic neural measures during the same cognitive task.

VR in humans has been used to assess performance on well-characterized spatial mazes previously used to investigate learning, memory and spatial navigation in rodents. For example, by combining VR with fMRI in humans, it is now possible to obtain a non-invasive measure of grid cells [72], previously reported using physiological recordings in rodents [188]. A similar approach has been used to ask whether the hippocampus represents 3D space, by combining VR with fMRI in humans [189,190] and comparing the data with physiological recordings acquired in rodents during spatial navigation in a comparable 3D environment [191].

However, VR in humans has been limited by traditional non-invasive imaging methods that require participants to remain motionless. With the introduction of scalp-mounted OPMs for acquisition of MEG data, it is now possible to obtain non-invasive measures of unconstrained head movement in humans [10]. When coupled with precise control of the background magnetic field, lightweight OPMs can be used to obtain MEG data as participants execute naturalistic movements within 3D VR [192]. This emerging technology provides a unique opportunity to directly compare neural measures of freely moving behaviour in humans with those obtained in animal models.

These VR behavioural assays may further bridge preclinical and clinical research as they are easy to translate into clinical populations. For example, performance on VR environments designed to mimic well-characterized spatial mazes previously investigated in rodents are sufficiently sensitive to detect clinical impairments observed in Alzheimer's disease [193] and schizophrenia [194]. Converting well-established behavioural paradigms into VR may, therefore, provide a means to compare data across species [195] and within patient populations [196].

In addition to VR, more complex behaviours can be captured by continuous monitoring via microchips and radio-frequency antennas or cameras [197–199]. In rodents, these measures can capture social hierarchies and exploration patterns, all in the ethologically valid—and potentially enriched—home environment, which in turn can be translated to equivalent human behaviours. For behaviours that cannot be readily modelled in rodents or other animal models, such as tool use, the complex behavioural repertoire of non-human primates provides a unique opportunity to model higher-order cognitive processes that are shared with humans.

## 8. Cross-species neural analyses: a common space

To integrate micro- and macroscopic levels of description, we must also take advantage of state-of-the-art tools available in different species (*Approach 3*). This necessitates cross-species comparison across different recording modalities using a common unit measure for neural activity.

Across different recording modalities, oscillatory dynamics provide a common signature of neural activity. Oscillations reflect changes in the amplitude and/or synchrony of transmembrane currents across a large number of neurons. They can be used to characterize the physiological state of a network or even predict neuronal spiking activity that shows phase-dependent excitability. The different classes of oscillation and their behavioural correlates appear broadly conserved throughout mammalian evolution [85]. Therefore, oscillatory dynamics recorded at the scalp using non-invasive methods, such as MEG in humans, can be directly related to invasive measures of the local field potential recorded in animal models. For example, when humans perform a spatial memory task in a virtual environment, theta frequency oscillations (6–10 Hz range) measured using MEG increase with virtual movement-onset [200], as observed using invasive electrophysiology in the hippocampus of both rodents [138,201] and epilepsy patients [202]. Similarly, gamma oscillations (30–70 Hz) measured in the human visual cortex using MEG [203] concur with invasive measures acquired in primate visual cortex [142].

**Figure 3.**
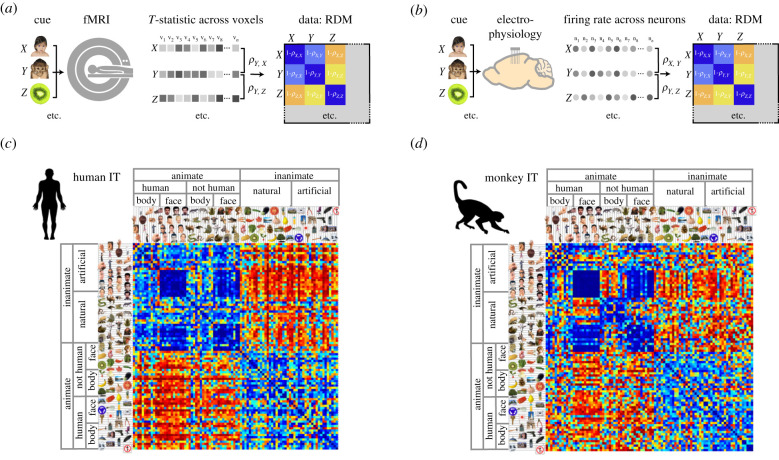
Cross-species neural analyses: RSA. RSA provides an analysis framework to compare data collected using multiple different recording methods. (*a*,*b*) RSA involves assessing the activity patterns across voxels (MRI) or across neurons (electrophysiology or calcium imaging) in response to different cues. The relative similarity between pairs of cue-specific activity patterns is then assessed using either a correlation or distance metric. The resulting metrics are entered into a representational dissimilarity matrix (RDM) to reveal the representational geometry of the data. (*c*,*d*) RSA applied to data from human and macaque monkey inferotemporal cortex (area IT) reveals striking similarities in the overall structure of representational information across species; adapted from [207].

While oscillatory brain dynamics provide a common signature for neuronal activity recorded across humans and animals, it is more challenging to relate non-invasive measures to spiking activity or synaptic processes. To translate between different recording modalities, we need to develop quantitative analytical approaches that assess shared features and deviations in anatomical and functional organization within a common space [180]. For anatomy, standardized templates are required to accurately assess coordinates within a common reference space [153]. For functional comparisons, a common data-analytical framework is called for. One possible approach involves extracting the representational geometry of a given brain region or neural circuit [204]. Building on mathematical literature on similarity analysis [205,206], this can be achieved using representational similarity analysis (RSA) (figure 3).

RSA involves estimating the relative similarity in multi-channel measures of neural activity between different conditions (e.g. stimuli or events). Therefore, for each pair of experimental conditions, the similarity in the response pattern elicited by the two conditions is assessed using a correlation or distance metric [208,209]. The resulting similarity measures for all pairs of conditions are then entered into a similarity matrix, where each cell in the matrix represents the similarity in neural activity between a pair of experimental conditions. In this manner, the similarity matrix describes the representational content carried by a given brain region (figure 3). This representational content can be quantified using the correlation distance between the similarity matrix and a theoretical model matrix, or by applying multi-dimensional scaling to the similarity matrix. RSA, therefore, provides a common framework to quantify the representational content of a given brain region across different recording modalities. Compared to other multivariate methods that aim to extract pattern information (such as multivariate pattern analysis), RSA is unique in abstracting the higher-order structure of representational information (second-order isomorphism) [204].

RSA has been successfully used to compare neural responses to visual objects in humans and non-human primates. Using fMRI and electrophysiological recordings, respectively, highly comparable representational structure can be observed in human and macaque inferotemporal cortex (area IT) [207] (figure 3). Similarly, RSA applied to fMRI data in humans and electrophysiological recordings in rodents reveals equivalent representational structure in the hippocampus on an inference task [210].

While this convergence between electrophysiology in animal models and multivariate human fMRI is encouraging, we must bear in mind the limitations of both fMRI and electrophysiology. As discussed above, for fMRI, the relationship between neural activity and the BOLD signal measured from a given voxel is non-trivial. For electrophysiology, only a biased subsample of neuronal responses are monitored and RSA overlooks information in the precise timing of spikes. The limitation of these recording modalities and differences in methodological sensitivity to representational information may give rise to differences in RSA or other multivariate methods employed across species. For example, multivariate pattern analysis applied to both fMRI and electrophysiology data from the macaque reveals that fMRI multivariate pattern analysis is insensitive to some representational information that can otherwise be decoded from single-unit recordings [211]. The accuracy of cross-species RSA will improve if we can account for the missing information inherent to each recording modality, which will be made apparent from investigations where multiple recording modalities are deployed in the same species (*Approach 1*).

Identifying spatial homologies between species as distant as the mouse and human presents a further challenge. The classic method of mapping like to like in anatomical ontologies, i.e. the mouse hippocampus is equal to the human hippocampus, remains the most employed method. Yet, it is likely that homologies between rodent and human will not be best captured by this type of one-to-one mapping. Instead, it is plausible that, over the course of evolution, functions that are highly localized in one species might be more distributed in another. Using additional information, such as the expression patterns of homologous genes or connectivity mapped via resting-state fMRI or diffusion MRI [212], could allow for more complex spatial transformations from one species to the other.

## 9. Cross-species computational modelling

In addition to analytical tools (see §8), computational models may be used to bridge the explanatory gap between neural recordings in humans and animal models (*Approaches 2* and *3*). By mathematically formalizing the complex interactions inherent to the brain, computational models can extract common quantitative descriptions for neural activity at both micro- and macroscopic levels. The resulting models may further be used to simulate and predict the effect of biophysical activity at both a cellular and systems level.

Perhaps the most elegant example of a computational model that provides a common description for neural activity at both the microscopic and macroscopic level comes from reinforcement learning algorithms. Based on animal learning experiments of classical conditioning [213,214], the Rescorla–Wagner algorithm was devised to account for the fact that learning is dependent upon the degree of unpredictability of a reinforcer [215,216]. The real-time extension of this algorithm, called temporal difference (TD) learning, incorporates a reward prediction error signal to learn a reward prediction signal. While this prediction error signal was initially hypothetical, researchers later discovered that it provides a good approximation for the temporal profile of activity in midbrain dopamine neurons, recorded using electrophysiology in the macaque [217,218] and in mice [89]. The TD learning algorithm can also be fit to human behaviour. When combined with fMRI, this model-based approach reveals a reliable signature of reward prediction error signals in the human midbrain during classical conditioning paradigms [219].

While computational models of reinforcement learning provide a compelling case study, their ability to successfully explain cellular and macroscopic descriptions of neural activity, together with behaviour, may be the exception rather than the norm. Such close correspondence between neural activity and algorithms that describe behaviour may simply be a rare find. More commonly, computational models fall short of such parsimonious mathematical abstraction, but may nevertheless constrain interpretation of data to provide hypothetical insight into the underlying circuit mechanism or predict brain responses to a set of stimuli.

For example, conceptual models, such as hippocampal models for pattern separation and completion, have explanatory power and constrain interpretation of data recorded at both a neural circuit level [220] and using human fMRI [75,221]. Biophysically plausible models inspired by invasive recording in animal models [222–225] can provide mechanistic insight into aggregate neural activity measured using non-invasive methods in humans [74,226–228]. More extensive network models, such as deep-neural networks trained using supervised learning, can account for visual representations in both the human and macaque brain [229]. In addition to performing image classification, extracting the internal representations of these deep-neural networks may inform our understanding of the mammalian visual cortex, holding predictive power for data acquired across different species.

Meanwhile, across biology, an alternative set of computational models are being developed to provide a means to directly translate findings across species. While avoiding the onerous task of biophysical realism, these models aim to explicitly translate findings from one species to another by describing a mapping between physiological parameters across species. Allometric scaling techniques can account for differences between species, where, for example, simple relationships between species are estimated using differences in body or brain weight. More accurate attempts to model physiological approaches have involved developing physiologically based pharmacokinetic (PBMK) modelling, where physiological and biochemical differences between species are used to translate mechanistic knowledge from one species into another [230–232]. These biophysical models are playing an increasingly important role in assessing the effects of potential therapeutic intervention across the biomedical sciences. This is critical for translational work where different phases of drug development are necessarily conducted in different species, and attrition rates for first-in-human studies are above 30% [233]. While currently used for translational work, these models may also provide the necessary tools for reliable cross-species extrapolation of basic research. Thus, by explicitly accounting for differences between species, computational models may formalize translation from microcircuit-level measures in animal models to macroscopic-level measures in humans.

## 10. Translational value of bridging the macroscopic and microscopic levels

Non-invasive measures of human brain activity are not routinely used as a tool for diagnosis, despite being readily available. As discussed above, this may be attributed to the explanatory gap between macroscopic measures of neural activity acquired using tools such as fMRI, and microcircuit mechanisms recorded in animal models.

Across medicine, this is perhaps most evident in modern psychiatry [234]. Diagnosis in psychiatry is still dependent upon subjective behavioural tests that are not linked with physiological or histological abnormalities. This is further complicated by poor delineation between disease categories and heterogeneity across the current disease classification schemes. But without an understanding of the underlying pathophysiology or the full complexity of psychiatric disease, assumptions made when selecting an animal disease model are compromised. Consequently, animal disease models often show limited predictive power and fail to translate to humans. The majority of neuropsychiatric drugs have instead been discovered serendipitously and the molecular targets largely reverse engineered [235].

Even in cases where there is a single gene disorder, promising results in animal models have at times failed to translate into drug development. A good example is the recent mGluR5 trials in Fragile X Syndrome. This high failure rate may in part be attributed to poor methodology. For example, animal studies appear to overestimate the likelihood of a treatment being effective, simply because negative results are often unpublished [236]. For disorders of brain development or ageing, a further challenge involves identifying common timepoints and stages of disease progression. Furthermore, despite highly conserved neuronal mechanisms via evolutionary descent, critical genetic, molecular, cellular and immunologic differences do occur between humans and animals. Therefore, animal models may provide a good model for a set of processes within a disease while failing to account for the full spectrum of physiological changes that occur in humans [237]. Critically, current measures in preclinical trials are often poorly translated to human clinical trials, providing a further translational challenge.

In the current socioeconomic climate, the cost of developing new neuropsychiatric drugs and neurotechnologies is rising, and as a result, pharmaceutical companies will move away from neuroscience to shift resources to more profitable areas. By developing a cross-species approach within fundamental neuroscience, we propose a means to build a foundation from which to bridge the explanatory gap between a behavioural characterization of neuropsychiatric disease and the underlying pathophysiology. This may be achieved by developing sensitive and effective tools for cross-species basic research that include imaging, behavioural assays, analytical methods and computational models, as outlined above.

## 11. Conclusion

Neuroscience has seen substantial development of non-invasive methods available for investigating the living human brain. Yet, owing to ethical and practical difficulties, these methods rarely permit insight into microcircuit-level mechanisms. To access the microcircuit, researchers instead rely on invasive recordings in animals, where recent advances in genetic tools now permit circuit-level manipulations with exquisite spatio-temporal precision. However, owing to challenges associated with animal research, there has been limited progress in understanding how neural circuits interact or relate to complex behaviour. Contemporary neuroscience thus faces an explanatory gap between macroscopic descriptions of cognition and behaviour in humans, and microscopic descriptions of cellular and synaptic processes in animal models. To close this explanatory gap and establish a more holistic description of brain function, here we call for an integrative cross-species approach. This approach is broadly justified by evidence showing preserved homology of neural circuits across mammals.

To embark on effective cross-species investigation, first we highlight the need to establish a deeper understanding of the relationship between non-invasive methods, such as the BOLD signal, and underlying neural activity. This may be achieved by employing multiple different tools within the same species. Second, to promote comparative investigation across species, we need to employ the same tools across multiple species. Cross-species MRI provides a unique opportunity to achieve this, by obtaining non-invasive markers of neural activity in both humans and animals that can be directly related to invasive manipulations in animals. When combined with cross-species behavioural assays, as exemplified by studies using VR, this comparative approach has the potential to reveal non-invasive markers of microcircuit mechanisms. Third, by taking advantage of the best tools available in each species, cross-species analyses and computational modelling may provide a means to translate measures of neural activity into a common space, despite differences in species and recording modality. Together, these three approaches may bridge the explanatory gap between macroscopic and microscopic descriptions of neural activity in the living human brain. In the context of clinical translation, where we have seen minimal success in neuropsychiatric drug development, a cross-species approach has the potential to reveal pathophysiology mechanisms responsible for neuropsychiatric disease.

## Data accessibility

This article has no additional data.

## References

[1] Hay M, Thomas DW, Craighead JL, Economides C, Rosenthal J 2014 Clinical development success rates for investigational drugs. Nat. Biotechnol. 32, 40–51. (10.1038/nbt.2786) 24406927

[2] Olesen J, Leonardi M 2003 The burden of brain diseases in Europe. Eur. J. Neurol. 10, 471–477. (10.1046/j.1468-1331.2003.00682.x) 12940825

[3] Hämäläinen M, Hari R, Ilmoniemi RJ, Knuutila J, Lounasmaa OV 1993 Magnetoencephalography—theory, instrumentation, and applications to noninvasive studies of the working human brain. Rev. Mod. Phys. 65, 413–497. (10.1103/RevModPhys.65.413)

[4] Gross J 2019 Magnetoencephalography in cognitive neuroscience: a primer. Neuron 104, 189–204. (10.1016/j.neuron.2019.07.001) 31647893

[5] Maess B, Koelsch S, Gunter TC, Friederici AD 2001 Musical syntax is processed in Broca's area: an MEG study. Nat. Neurosci. 4, 540–545. (10.1038/87502) 11319564

[6] Houde JF, Nagarajan SS, Sekihara K, Merzenich MM 2002 Modulation of the auditory cortex during speech: an MEG study. J. Cogn. Neurosci. 14, 1125–1138. (10.1162/089892902760807140) 12495520

[7] Fuentemilla L, Penny WD, Cashdollar N, Bunzeck N, Düzel E 2010 Theta-coupled periodic replay in working memory. Curr. Biol. 20, 606–612. (10.1016/j.cub.2010.01.057) 20303266 PMC2856918

[8] Jafarpour A, Fuentemilla L, Horner AJ, Penny W, Duzel E 2014 Replay of very early encoding representations during recollection. J. Neurosci. 34, 242–248. (10.1523/JNEUROSCI.1865-13.2014) 24381285 PMC3866486

[9] Borna A et al. 2017 A 20-channel magnetoencephalography system based on optically pumped magnetometers. Phys. Med. Biol. 62, 8909–8923. (10.1088/1361-6560/aa93d1) 29035875 PMC5890515

[10] Boto E et al. 2018 Moving magnetoencephalography towards real-world applications with a wearable system. Nature 555, 657–661. (10.1038/nature26147) 29562238 PMC6063354

[11] Johnson CN, Schwindt PDD, Weisend M 2013 Multi-sensor magnetoencephalography with atomic magnetometers. Phys. Med. Biol. 58, 6065–6077. (10.1088/0031-9155/58/17/6065) 23939051 PMC4030549

[12] Kamada K, Sato D, Ito Y, Natsukawa H, Okano K, Mizutani N, Kobayashi T 2015 Human magnetoencephalogram measurements using newly developed compact module of high-sensitivity atomic magnetometer. Jpn J. Appl. Phys. 54, 026601 (10.7567/JJAP.54.026601)

[13] Boto E et al. 2017 A new generation of magnetoencephalography: room temperature measurements using optically-pumped magnetometers. NeuroImage 149, 404–414. (10.1016/j.neuroimage.2017.01.034) 28131890 PMC5562927

[14] Logothetis NK, Wandell BA 2004 Interpreting the BOLD signal. Annu. Rev. Physiol. 66, 735–769. (10.1146/annurev.physiol.66.082602.092845) 14977420

[15] Logothetis NK 2003 The underpinnings of the BOLD functional magnetic resonance imaging signal. J. Neurosci. 23, 3963–3971. (10.1523/JNEUROSCI.23-10-03963.2003) 12764080 PMC6741096

[16] Brinker G, Bock C, Busch E, Krep H, Hossmann K-A, Hoehn-Berlage M 1999 Simultaneous recording of evoked potentials and T-weighted MR images during somatosensory stimulation of rat. Magn. Reson. Med. 41, 469–473. (10.1002/(SICI)1522-2594(199903)41:3<469::AID-MRM7>3.0.CO;2-9) 10204868

[17] Mathiesen C, Caesar K, Akgören N, Lauritzen M 1998 Modification of activity-dependent increases of cerebral blood flow by excitatory synaptic activity and spikes in rat cerebellar cortex. J. Physiol. 512, 555–566. (10.1111/j.1469-7793.1998.555be.x) 9763643 PMC2231204

[18] Ogawa S, Lee T-M, Stepnoski R, Chen W, Zhu X-H, Ugurbil K 2000 An approach to probe some neural systems interaction by functional MRI at neural time scale down to milliseconds. Proc. Natl Acad. Sci. USA 97, 11 026–11 031. (10.1073/pnas.97.20.11026) PMC27142 11005873

[19] Hyder F, Rothman DL, Shulman RG 2002 Total neuroenergetics support localized brain activity: implications for the interpretation of fMRI. Proc. Natl Acad. Sci. USA 99, 10 771–10 776. (10.1073/pnas.132272299) 12134057 PMC125040

[20] Rees G, Friston K, Koch C 2000 A direct quantitative relationship between the functional properties of human and macaque V5. Nat. Neurosci. 3, 716–723. (10.1038/76673) 10862705

[21] Smith AJ, Blumenfeld H, Behar KL, Rothman DL, Shulman RG, Hyder F 2002 Cerebral energetics and spiking frequency: the neurophysiological basis of fMRI. Proc. Natl Acad. Sci. USA 99, 10 765–10 770. (10.1073/pnas.132272199) 12134056 PMC125038

[22] Sheth SA, Nemoto M, Guiou M, Walker M, Pouratian N, Toga AW 2004 Linear and nonlinear relationships between neuronal activity, oxygen metabolism, and hemodynamic responses. Neuron 42, 347–355. (10.1016/S0896-6273(04)00221-1) 15091348

[23] Hoffmann MB, Stadler J, Kanowski M, Speck O 2009 Retinotopic mapping of the human visual cortex at a magnetic field strength of 7T. Clin. Neurophysiol. 120, 108–116. (10.1016/j.clinph.2008.10.153) 19071059

[24] Olman CA, Van de Moortele P-F, Schumacher JF, Guy JR, Uğurbil K, Yacoub E 2010 Retinotopic mapping with spin echo BOLD at 7T. Magn. Reson. Imaging 28, 1258–1269. (10.1016/j.mri.2010.06.001) 20656431 PMC2963715

[25] Sánchez-Panchuelo RM, Francis ST, Schluppeck D, Bowtell RW 2012 Correspondence of human visual areas identified using functional and anatomical MRI *in vivo* at 7 T. J. Magn. Reson. Imaging 35, 287–299. (10.1002/jmri.22822) 21964755

[26] Costa SD, van der Zwaag W, Marques JP, Frackowiak RSJ, Clarke S, Saenz M 2011 Human primary auditory cortex follows the shape of Heschl's gyrus. J. Neurosci. 31, 14 067–14 075. (10.1523/JNEUROSCI.2000-11.2011) PMC6623669 21976491

[27] Formisano E, Kim D-S, Di Salle F, van de Moortele P-F, Ugurbil K, Goebel R 2003 Mirror-symmetric tonotopic maps in human primary auditory cortex. Neuron 40, 859–869. (10.1016/S0896-6273(03)00669-X) 14622588

[28] Martuzzi R, van der Zwaag W, Farthouat J, Gruetter R, Blanke O 2014 Human finger somatotopy in areas 3b, 1, and 2: a 7T fMRI study using a natural stimulus. Hum. Brain Mapp. 35, 213–226. (10.1002/hbm.22172) 22965769 PMC6869627

[29] Haak KV, Marquand AF, Beckmann CF 2018 Connectopic mapping with resting-state fMRI. NeuroImage 15, 83–94. (10.1016/j.neuroimage.2017.06.075) 28666880

[30] Marquand AF, Haak KV, Beckmann CF 2017 Functional corticostriatal connection topographies predict goal directed behaviour in humans. Nat. Hum. Behav. 1, 0146 (10.1038/s41562-017-0146). 28804783 PMC5549843

[31] Kikkert S, Kolasinski J, Jbabdi S, Tracey I, Beckmann CF, Johansen-Berg H, Makin TR 2016 Revealing the neural fingerprints of a missing hand. eLife 5, e15292 (10.7554/eLife.15292) 27552053 PMC5040556

[32] Fracasso A, Koenraads Y, Porro GL, Dumoulin SO 2016 Bilateral population receptive fields in congenital hemihydranencephaly. Ophthal. Physiol. Opt. 36, 324–334. (10.1111/opo.12294) 27112226

[33] Hoffmann MB et al. 2012 Plasticity and stability of the visual system in human achiasma. Neuron 75, 393–401. (10.1016/j.neuron.2012.05.026) 22884323 PMC3427398

[34] Cheng K, Waggoner RA, Tanaka K 2001 Human ocular dominance columns as revealed by high-field functional magnetic resonance imaging. Neuron 32, 359–374. (10.1016/S0896-6273(01)00477-9) 11684004

[35] Yacoub E, Shmuel A, Logothetis N, Uğurbil K 2007 Robust detection of ocular dominance columns in humans using Hahn Spin Echo BOLD functional MRI at 7 Tesla. NeuroImage 37, 1161–1177. (10.1016/j.neuroimage.2007.05.020) 17702606 PMC2040323

[36] Shipp S 2007 Structure and function of the cerebral cortex. Curr. Biol. 17, R443–R449. (10.1016/j.cub.2007.03.044) 17580069

[37] Koopmans PJ, Barth M, Norris DG 2010 Layer-specific BOLD activation in human V1. Hum. Brain Mapp. 31, 1297–1304. (10.1002/hbm.20936) 20082333 PMC6870878

[38] Lawrence SJD, Formisano E, Muckli L, de Lange FP 2019 Laminar fMRI: applications for cognitive neuroscience. NeuroImage 197, 785–791. (10.1016/j.neuroimage.2017.07.004) 28687519

[39] Dumoulin SO, Fracasso A, van der Zwaag W, Siero JCW, Petridou N 2018 Ultra-high field MRI: advancing systems neuroscience towards mesoscopic human brain function. NeuroImage 168, 345–357. (10.1016/j.neuroimage.2017.01.028) 28093360

[40] Harris KD, Mrsic-Flogel TD 2013 Cortical connectivity and sensory coding. Nature 503, 51–58. (10.1038/nature12654) 24201278

[41] Anderson JC, Martin KAC 2009 The synaptic connections between cortical areas V1 and V2 in macaque monkey. J. Neurosci. 29, 11 283–11 293. (10.1523/JNEUROSCI.5757-08.2009) PMC6665918 19741135

[42] Rockland KS, Virga A 1989 Terminal arbors of individual ‘Feedback’ axons projecting from area V2 to V1 in the macaque monkey: a study using immunohistochemistry of anterogradely transported *Phaseolus vulgaris*-leucoagglutinin. J. Comp. Neurol. 285, 54–72. (10.1002/cne.902850106) 2754047

[43] Kok P, Bains LJ, van Mourik T, Norris DG, de Lange FP 2016 Selective activation of the deep layers of the human primary visual cortex by top–down feedback. Curr. Biol. 26, 371–376. (10.1016/j.cub.2015.12.038) 26832438

[44] Muckli L, De Martino F, Vizioli L, Petro LS, Smith FW, Ugurbil K, Goebel R, Yacoub E 2015 Contextual feedback to superficial layers of V1. Curr. Biol. 25, 2690–2695. (10.1016/j.cub.2015.08.057) 26441356 PMC4612466

[45] Fukuda M, Moon C-H, Wang P, Kim S-G 2006 Mapping iso-orientation columns by contrast agent-enhanced functional magnetic resonance imaging: reproducibility, specificity, and evaluation by optical imaging of intrinsic signal. J. Neurosci. 26, 11 821–11 832. (10.1523/JNEUROSCI.3098-06.2006) PMC6674871 17108155

[46] Kim D-S, Duong TQ, Kim S-G 2000 High-resolution mapping of iso-orientation columns by fMRI. Nat. Neurosci. 3, 164–169. (10.1038/72109) 10649572

[47] Moon C-H, Fukuda M, Park S-H, Kim S-G 2007 Neural interpretation of blood oxygenation level-dependent fMRI maps at submillimeter columnar resolution. J. Neurosci. 27, 6892–6902. (10.1523/JNEUROSCI.0445-07.2007) 17596437 PMC6672231

[48] Zhao F, Wang P, Hendrich K, Kim S-G 2005 Spatial specificity of cerebral blood volume-weighted fMRI responses at columnar resolution. NeuroImage 27, 416–424. (10.1016/j.neuroimage.2005.04.011) 15923128

[49] Self MW, van Kerkoerle T, Supèr H, Roelfsema PR 2013 Distinct roles of the cortical layers of area V1 in figure-ground segregation. Curr. Biol. 23, 2121–2129. (10.1016/j.cub.2013.09.013) 24139742

[50] Takahashi N, Oertner TG, Hegemann P, Larkum ME 2016 Active cortical dendrites modulate perception. Science 354, 1587–1590. (10.1126/science.aah6066) 28008068

[51] Uludağ K, Blinder P 2018 Linking brain vascular physiology to hemodynamic response in ultra-high field MRI. NeuroImage 168, 279–295. (10.1016/j.neuroimage.2017.02.063) 28254456

[52] Barth M, Breuer F, Koopmans PJ, Norris DG, Poser BA 2016 Simultaneous multislice (SMS) imaging techniques. Magn. Reson. Med. 75, 63–81. (10.1002/mrm.25897) 26308571 PMC4915494

[53] Feinberg DA, Yacoub E 2012 The rapid development of high speed, resolution and precision in fMRI. NeuroImage 62, 720–725. (10.1016/j.neuroimage.2012.01.049) 22281677 PMC3389295

[54] Setsompop K, Feinberg DA, Polimeni JR 2016 Rapid brain MRI acquisition techniques at ultra-high fields. NMR Biomed. 29, 1198–1221. (10.1002/nbm.3478) 26835884 PMC5245168

[55] Henson RNA 2004 Analysis of fMRI time series: linear time-invariant models, event-related fMRI and optimal experimental design In *Human brain function* (eds RSJ Frackowiak, KJ Friston, CD Frith, RJ Dolan, CJ Price, S Zeki, J Ashburner, WD Penny), pp. 793–822, 2nd edn. Amsterdam, The Netherlands: Elsevier (10.1016/B978-012264841-0/50042-1)

[56] Josephs O, Turner R, Friston K 1997 Event-related fMRI. Hum. Brain Mapp. 5, 243–248. (10.1002/(SICI)1097-0193(1997)5:4<243::AID-HBM7>3.0.CO;2-3) 20408223

[57] Friston KJ, Fletcher P, Josephs O, Holmes A, Rugg MD, Turner R 1998 Event-related fMRI: characterizing differential responses. NeuroImage 7, 30–40. (10.1006/nimg.1997.0306) 9500830

[58] Friston KJ, Josephs O, Rees G, Turner R 1998 Nonlinear event-related responses in fMRI. Magn. Reson. Med. 39, 41–52. (10.1002/mrm.1910390109) 9438436

[59] Friston KJ, Mechelli A, Turner R, Price CJ 2000 Nonlinear responses in fMRI: the balloon model, Volterra kernels, and other hemodynamics. NeuroImage 12, 466–477. (10.1006/nimg.2000.0630) 10988040

[60] Schuck NW, Niv Y 2019 Sequential replay of nonspatial task states in the human hippocampus. Science 364, eaaw5181 (10.1126/science.aaw5181) 31249030 PMC7241311

[61] Kurth-Nelson Z, Economides M, Dolan RJ, Dayan P 2016 Fast sequences of non-spatial state representations in humans. Neuron 91, 194–204. (10.1016/j.neuron.2016.05.028) 27321922 PMC4942698

[62] Liu Y, Dolan RJ, Kurth-Nelson Z, Behrens TEJ 2019 Human replay spontaneously reorganizes experience. Cell 178, 640–652.e14. (10.1016/j.cell.2019.06.012) 31280961 PMC6657653

[63] Louie K, Wilson MA 2001 Temporally structured replay of awake hippocampal ensemble activity during rapid eye movement sleep. Neuron 29, 145–156. (10.1016/S0896-6273(01)00186-6) 11182087

[64] Wilson MA, McNaughton BL 1994 Reactivation of hippocampal ensemble memories during sleep. Science 265, 676–679. (10.1126/science.8036517) 8036517

[65] Nádasdy Z, Hirase H, Czurkó A, Csicsvari J, Buzsáki G 1999 Replay and time compression of recurring spike sequences in the hippocampus. J. Neurosci. 19, 9497–9507. (10.1523/JNEUROSCI.19-21-09497.1999) 10531452 PMC6782894

[66] Foster DJ 2017 Replay comes of age. Annu. Rev. Neurosci. 40, 581–602. (10.1146/annurev-neuro-072116-031538) 28772098

[67] Joo HR, Frank LM 2018 The hippocampal sharp wave–ripple in memory retrieval for immediate use and consolidation. Nat. Rev. Neurosci. 19, 744–757. (10.1038/s41583-018-0077-1) 30356103 PMC6794196

[68] Buzsáki G 2015 Hippocampal sharp wave-ripple: a cognitive biomarker for episodic memory and planning. Hippocampus 25, 1073–1188. (10.1002/hipo.22488) 26135716 PMC4648295

[69] Logothetis NK 2008 What we can do and what we cannot do with fMRI. Nature 453, 869–878. (10.1038/nature06976) 18548064

[70] Devor A et al. 2007 Suppressed neuronal activity and concurrent arteriolar vasoconstriction may explain negative blood oxygenation level-dependent signal. J. Neurosci. 27, 4452–4459. (10.1523/JNEUROSCI.0134-07.2007) 17442830 PMC2680207

[71] Uhlirova H et al. 2016 Cell type specificity of neurovascular coupling in cerebral cortex. eLife 5, e14315 (10.7554/eLife.14315) 27244241 PMC4933561

[72] Doeller CF, Barry C, Burgess N 2010 Evidence for grid cells in a human memory network. Nature 463, 657–661. (10.1038/nature08704) 20090680 PMC3173857

[73] Kolasinski J, Makin TR, Jbabdi S, Clare S, Stagg CJ, Johansen-Berg H 2016 Investigating the stability of fine-grain digit somatotopy in individual human participants. J. Neurosci. 36, 1113–1127. (10.1523/JNEUROSCI.1742-15.2016) 26818501 PMC4728720

[74] Barron HC, Vogels TP, Emir UE, Makin TR, O'Shea J, Clare S, Jbabdi S, Dolan RJ, Behrens TEJ 2016 Unmasking latent inhibitory connections in human cortex to reveal dormant cortical memories. Neuron 90, 191–203. (10.1016/j.neuron.2016.02.031) 26996082 PMC4826438

[75] Koolschijn RS, Emir UE, Pantelides AC, Nili H, Behrens TEJ, Barron HC 2018 The hippocampus and neocortical inhibitory engrams protect against memory interference. Neuron 101, 528–541. (10.1016/j.neuron.2018.11.042) 30581011 PMC6560047

[76] Zhang N, Zhu X-H, Yacoub E, Ugurbil K, Chen W 2010 Functional MRI mapping neuronal inhibition and excitation at columnar level in human visual cortex. Exp. Brain Res. 204, 515–524. (10.1007/s00221-010-2318-z) 20571785 PMC2937580

[77] Ekstrom AD, Kahana MJ, Caplan JB, Fields TA, Isham EA, Newman EL, Fried I 2003 Cellular networks underlying human spatial navigation. Nature 425, 184–188. (10.1038/nature01964) 12968182

[78] Fell J, Klaver P, Lehnertz K, Grunwald T, Schaller C, Elger CE, Fernández G 2001 Human memory formation is accompanied by rhinal–hippocampal coupling and decoupling. Nat. Neurosci. 4, 1259–1264. (10.1038/nn759) 11694886

[79] Jun JJ et al. 2017 Fully integrated silicon probes for high-density recording of neural activity. Nature 551, 232–236. (10.1038/nature24636) 29120427 PMC5955206

[80] Steinmetz NA, Zatka-Haas P, Carandini M, Harris KD 2019 Distributed coding of choice, action and engagement across the mouse brain. Nature 576, 266–273. (10.1038/s41586-019-1787-x) 31776518 PMC6913580

[81] Chung JE et al. 2019 High-density, long-lasting, and multi-region electrophysiological recordings using polymer electrode arrays. Neuron 101, 21–31.e5. (10.1016/j.neuron.2018.11.002) 30502044 PMC6326834

[82] Buccino AP, Hurwitz CL, Garcia S, Magland J, Siegle JH, Hurwitz R, Hennig MH 2020 SpikeInterface, a unified framework for spike sorting. *bioRxiv* 796599 (10.1101/796599) PMC7704107 33170122

[83] Magland J, Jun JJ, Lovero E, Morley AJ, Hurwitz CL, Buccino AP, Garcia S, Barnett AH 2020 SpikeForest, reproducible web-facing ground-truth validation of automated neural spike sorters. Elife 9, e55167 (10.7554/eLife.55167) 32427564 PMC7237210

[84] Berényi A et al. 2014 Large-scale, high-density (up to 512 channels) recording of local circuits in behaving animals. J. Neurophysiol. 111, 1132–1149. (10.1152/jn.00785.2013) 24353300 PMC3949233

[85] Buzsáki G, Draguhn A 2004 Neuronal oscillations in cortical networks. Science 304, 1926–1929. (10.1126/science.1099745) 15218136

[86] Fries P 2015 Rhythms for cognition: communication through coherence. Neuron 88, 220–235. (10.1016/j.neuron.2015.09.034) 26447583 PMC4605134

[87] Somogyi P, Tamás G, Lujan R, Buhl EH 1998 Salient features of synaptic organisation in the cerebral cortex. Brain Res. Brain Res. Rev. 26, 113–135. (10.1016/S0165-0173(97)00061-1) 9651498

[88] Monyer H, Markram H 2004 Interneuron diversity series: molecular and genetic tools to study GABAergic interneuron diversity and function. Trends Neurosci. 27, 90–97. (10.1016/j.tins.2003.12.008) 15102488

[89] Cohen JY, Haesler S, Vong L, Lowell BB, Uchida N 2012 Neuron-type-specific signals for reward and punishment in the ventral tegmental area. Nature 482, 85–88. (10.1038/nature10754) 22258508 PMC3271183

[90] Csicsvari J, Hirase H, Czurkó A, Mamiya A, Buzsáki G 1999 Oscillatory coupling of hippocampal pyramidal cells and interneurons in the behaving rat. J. Neurosci. 19, 274–287. (10.1523/JNEUROSCI.19-01-00274.1999) 9870957 PMC6782375

[91] Lapray D et al. 2012 Behavior-dependent specialization of identified hippocampal interneurons. Nat. Neurosci. 15, 1265–1271. (10.1038/nn.3176) 22864613 PMC3433735

[92] Luo L, Callaway EM, Svoboda K 2018 Genetic dissection of neural circuits: a decade of progress. Neuron 98, 865 (10.1016/j.neuron.2018.05.004) 29772206 PMC5997456

[93] Kerr JND, Denk W 2008 Imaging *in vivo*: watching the brain in action. Nat. Rev. Neurosci. 9, 195–205. (10.1038/nrn2338) 18270513

[94] Runyan CA, Schummers J, Van Wart A, Kuhlman SJ, Wilson NR, Huang ZJ, Sur M 2010 Response features of parvalbumin-expressing interneurons suggest precise roles for subtypes of inhibition in visual cortex. Neuron 67, 847–857. (10.1016/j.neuron.2010.08.006) 20826315 PMC2948796

[95] Hofer SB, Ko H, Pichler B, Vogelstein J, Ros H, Zeng H, Lein E, Lesica NA, Mrsic-Flogel TD 2011 Differential connectivity and response dynamics of excitatory and inhibitory neurons in visual cortex. Nat. Neurosci. 14, 1045–1052. (10.1038/nn.2876) 21765421 PMC6370002

[96] Prevedel R et al. 2014 Simultaneous whole-animal 3D imaging of neuronal activity using light-field microscopy. Nat. Methods 11, 727–730. (10.1038/nmeth.2964) 24836920 PMC4100252

[97] Ahrens MB, Orger MB, Robson DN, Li JM, Keller PJ 2013 Whole-brain functional imaging at cellular resolution using light-sheet microscopy. Nat. Methods 10, 413–420. (10.1038/nmeth.2434) 23524393

[98] Portugues R, Feierstein CE, Engert F, Orger MB 2014 Whole-brain activity maps reveal stereotyped, distributed networks for visuomotor behavior. Neuron 81, 1328–1343. (10.1016/j.neuron.2014.01.019) 24656252 PMC4448760

[99] Hsu K-J, Lin Y-Y, Chiang A-S, Chu S-W 2018 Whole-brain imaging and characterization of *Drosophila* brains based on one-, two-, and three-photon excitations. *bioRxiv* 339531 (10.1101/339531)

[100] Lin MZ, Schnitzer MJ 2016 Genetically encoded indicators of neuronal activity. Nat. Neurosci. 19, 1142–1153. (10.1038/nn.4359) 27571193 PMC5557009

[101] Häusser M 2014 Optogenetics: the age of light. Nat. Methods 11, 1012–1014. (10.1038/nmeth.3111) 25264778

[102] Deisseroth K 2011 Optogenetics. Nat. Methods 8, 26–29. (10.1038/nmeth.f.324) 21191368 PMC6814250

[103] Hubel DH, Wiesel TN 1977 Ferrier lecture - Functional architecture of macaque monkey visual cortex. Proc. R. Soc. Lond. B 198, 1–59. (10.1098/rspb.1977.0085) 20635

[104] Buzsáki G 2004 Large-scale recording of neuronal ensembles. Nat. Neurosci. 7, 446–451. (10.1038/nn1233) 15114356

[105] Datta SR, Anderson DJ, Branson K, Perona P, Leifer A 2019 Computational neuroethology: a call to action. Neuron 104, 11–24. (10.1016/j.neuron.2019.09.038) 31600508 PMC6981239

[106] Anderson DJ, Perona P 2014 Toward a science of computational ethology. Neuron 84, 18–31. (10.1016/j.neuron.2014.09.005) 25277452

[107] Horton JC, Adams DL 2005 The cortical column: a structure without a function. Phil. Trans. R. Soc. B. 360, 837–862. (10.1098/rstb.2005.1623) 15937015 PMC1569491

[108] DeFelipe J, Alonso-Nanclares L, Arellano JI 2002 Microstructure of the neocortex: comparative aspects. J. Neurocytol. 31, 299–316. (10.1023/A:1024130211265) 12815249

[109] Mouse Genome Sequencing Consortium. 2002 Initial sequencing and comparative analysis of the mouse genome. Nature 420, 520–562. (10.1038/nature01262) 12466850

[110] Krubitzer L 2007 The magnificent compromise: cortical field evolution in mammals. Neuron 56, 201–208. (10.1016/j.neuron.2007.10.002) 17964240

[111] Zeng H et al. 2012 Large-scale cellular-resolution gene profiling in human neocortex reveals species-specific molecular signatures. Cell 149, 483–496. (10.1016/j.cell.2012.02.052) 22500809 PMC3328777

[112] Brodmann K 1905 Beiträge zur histologischen Lokalisation der Grosshirnrinde. III. Der Rindenfelder der niederen Affen. J. Physiol. Neurol. 4, 177–226.

[113] DeFelipe J 1997 Types of neurons, synaptic connections and chemical characteristics of cells immunoreactive for calbindin-D28 K, parvalbumin and calretinin in the neocortex. J. Chem. Neuroanat. 14, 1–19. (10.1016/S0891-0618(97)10013-8) 9498163

[114] Xu X, Roby KD, Callaway EM 2006 Mouse cortical inhibitory neuron type that coexpresses somatostatin and calretinin. J. Comp. Neurol. 499, 144–160. (10.1002/cne.21101) 16958092

[115] Cauli B, Zhou X, Tricoire L, Toussay X, Staiger JF 2014 Revisiting enigmatic cortical calretinin-expressing interneurons. Front. Neuroanat. 8 [cited 11 December 2019]. See https://www.frontiersin.org/articles/10.3389/fnana.2014.00052/full (10.3389/fnana.2014.00052) PMC4067953 25009470

[116] Varga C, Tamas G, Barzo P, Olah S, Somogyi P 2015 Molecular and electrophysiological characterization of GABAergic interneurons expressing the transcription factor COUP-TFII in the adult human temporal cortex. Cereb. Cortex. 25, 4430–4449. (10.1093/cercor/bhv045) 25787832 PMC4768361

[117] DeFelipe J et al. 2013 New insights into the classification and nomenclature of cortical GABAergic interneurons. Nat. Rev. Neurosci. 14, 202–216. (10.1038/nrn3444) 23385869 PMC3619199

[118] Szabadics J, Varga C, Molnár G, Oláh S, Barzó P, Tamás G 2006 Excitatory effect of GABAergic axo-axonic cells in cortical microcircuits. Science 311, 233–235. (10.1126/science.1121325) 16410524

[119] Kaas JH, Collins CE 2001 The organization of sensory cortex. Curr. Opin. Neurobiol. 11, 498–504. (10.1016/S0959-4388(00)00240-3) 11502398

[120] Rilling JK, Glasser MF, Preuss TM, Ma X, Zhao T, Hu X, Behrens TEJ 2008 The evolution of the arcuate fasciculus revealed with comparative DTI. Nat. Neurosci. 11, 426–428. (10.1038/nn2072) 18344993

[121] Krubitzer L, Kaas J 2005 The evolution of the neocortex in mammals: how is phenotypic diversity generated? Curr. Opin Neurobiol. 15, 444–453. (10.1016/j.conb.2005.07.003) 16026978

[122] Eichert N, Robinson EC, Bryant KL, Jbabdi S, Jenkinson M, Li L, Krug K, Watkins KE, Mars RB 2019 Cross-species cortical alignment identifies different types of neuroanatomical reorganization in higher primates. *bioRxiv*, 645234 (10.7554/eLife.53232) PMC7180052 32202497

[123] Vincent JL et al. 2007 Intrinsic functional architecture in the anaesthetized monkey brain. Nature 447, 83–86. (10.1038/nature05758) 17476267

[124] Rilling JK, Barks SK, Parr LA, Preuss TM, Faber TL, Pagnoni G, Bremner JD, Votaw JR 2007 A comparison of resting-state brain activity in humans and chimpanzees. Proc. Natl Acad. Sci. USA 104, 17 146–17 151. (10.1073/pnas.0705132104) PMC2040430 17940032

[125] Li L, Hu X, Preuss TM, Glasser MF, Damen FW, Qiu Y, Rilling J 2013 Mapping putative hubs in human, chimpanzee and rhesus macaque connectomes via diffusion tractography. NeuroImage 80, 462–474. (10.1016/j.neuroimage.2013.04.024) 23603286 PMC3720835

[126] Vanduffel W, Zhu Q, Orban GA 2014 Monkey cortex through fMRI glasses. Neuron 83, 533–550. (10.1016/j.neuron.2014.07.015) 25102559 PMC4698430

[127] Peeters R, Simone L, Nelissen K, Fabbri-Destro M, Vanduffel W, Rizzolatti G, Orban GA 2009 The representation of tool use in humans and monkeys: common and uniquely human features. J. Neurosci. 29, 11 523–11 539. (10.1523/JNEUROSCI.2040-09.2009) PMC6665774 19759300

[128] Wilson B et al. 2015 Auditory sequence processing reveals evolutionarily conserved regions of frontal cortex in macaques and humans. Nat. Commun. 6, 8901 (10.1038/ncomms9901) 26573340 PMC4660034

[129] Chau BKH, Sallet J, Papageorgiou GK, Noonan MP, Bell AH, Walton ME, Rushworth MFS 2015 Contrasting roles for orbitofrontal cortex and amygdala in credit assignment and learning in macaques. Neuron 87, 1106–1118. (10.1016/j.neuron.2015.08.018) 26335649 PMC4562909

[130] Felleman DJ, Van Essen DC 1991 Distributed hierarchical processing in the primate cerebral cortex. Cereb. Cortex. 1, 1–47. (10.1093/cercor/1.1.1) 1822724

[131] O'Keefe J, Dostrovsky J 1971 The hippocampus as a spatial map. Preliminary evidence from unit activity in the freely-moving rat. Brain Res. 34, 171–175. (10.1016/0006-8993(71)90358-1) 5124915

[132] McHugh TJ, Blum KI, Tsien JZ, Tonegawa S, Wilson MA 1996 Impaired hippocampal representation of space in CA1-specific NMDAR1 knockout mice. Cell 87, 1339–1349. (10.1016/S0092-8674(00)81828-0) 8980239

[133] Muir GM, Brown JE, Carey JP, Hirvonen TP, Santina CCD, Minor LB, Taube JS 2009 Disruption of the head direction cell signal after occlusion of the semicircular canals in the freely moving chinchilla. J. Neurosci. 29, 14 521–14 533. (10.1523/JNEUROSCI.3450-09.2009) 19923286 PMC2821030

[134] Ulanovsky N, Moss CF 2007 Hippocampal cellular and network activity in freely moving echolocating bats. Nat. Neurosci. 10, 224–233. (10.1038/nn1829) 17220886

[135] Ono T, Nakamura K, Nishijo H, Eifuku S 1993 Monkey hippocampal neurons related to spatial and nonspatial functions. J. Neurophysiol. 70, 1516–1529. (10.1152/jn.1993.70.4.1516) 8283212

[136] O'Keefe J, Burgess N 1996 Geometric determinants of the place fields of hippocampal neurons. Nature 381, 425–428. (10.1038/381425a0) 8632799

[137] Las L, Ulanovsky N 2014 Hippocampal neurophysiology across species. In Space, time and memory in the hippocampal formation (eds Derdikman D, Knierim JJ), pp. 431–461. Vienna, Austria: Springer [cited 8 December 2019] (10.1007/978-3-7091-1292-2_16)

[138] O'Keefe J, Nadel L 1978 The hippocampus as a cognitive map. Oxford, UK: Clarendon Press.

[139] Aboitiz F, López J, Montiel J 2003 Long distance communication in the human brain: timing constraints for inter-hemispheric synchrony and the origin of brain lateralization. Biol. Res. 36, 89–99. (10.4067/S0716-97602003000100007) 12795208

[140] Wang SS-H, Shultz JR, Burish MJ, Harrison KH, Hof PR, Towns LC, Wagers MW, Wyatt KD 2008 Functional trade-offs in white matter axonal scaling. J. Neurosci. 28, 4047–4056. (10.1523/JNEUROSCI.5559-05.2008) 18400904 PMC2779774

[141] Mars RB, Eichert N, Jbabdi S, Verhagen L, Rushworth MFS 2018 Connectivity and the search for specializations in the language-capable brain. Curr. Opin. Behav. Sci. 21, 19–26. (10.1016/j.cobeha.2017.11.001) PMC7610656 33898657

[142] Logothetis NK, Pauls J, Augath M, Trinath T, Oeltermann A 2001 Neurophysiological investigation of the basis of the fMRI signal. Nature 412, 150–157. (10.1038/35084005) 11449264

[143] Pan WJ, Thompson G, Magnuson M, Majeed W, Jaeger D, Keilholz S 2010 Simultaneous fMRI and electrophysiology in the rodent brain. JoVE (J. Visualized Exp.) 42, e1901 (10.3791/1901) PMC3156003 20811324

[144] Magri C, Schridde U, Murayama Y, Panzeri S, Logothetis NK 2012 The amplitude and timing of the BOLD signal reflects the relationship between local field potential power at different frequencies. J. Neurosci. 32, 1395–1407. (10.1523/JNEUROSCI.3985-11.2012) 22279224 PMC6796252

[145] Ma Y, Shaik MA, Kozberg MG, Kim SH, Portes JP, Timerman D, Hillman EMC 2016 Resting-state hemodynamics are spatiotemporally coupled to synchronized and symmetric neural activity in excitatory neurons. Proc. Natl Acad. Sci. USA 113, E8463–E8471. (10.1073/pnas.1525369113) 27974609 PMC5206542

[146] Hillman EMC 2014 Coupling mechanism and significance of the BOLD signal: a status report. Annu. Rev. Neurosci. 37, 161–181. (10.1146/annurev-neuro-071013-014111) 25032494 PMC4147398

[147] Rilling JK 2014 Comparative primate neuroimaging: insights into human brain evolution. Trends Cogn. Sci. 18, 46–55. (10.1016/j.tics.2013.09.013) 24501779

[148] Reid AT, Lewis J, Bezgin G, Khundrakpam B, Eickhoff SB, McIntosh AR, Bellec P, Evans AC 2016 A cross-modal, cross-species comparison of connectivity measures in the primate brain. NeuroImage 125, 311–331. (10.1016/j.neuroimage.2015.10.057) 26515902

[149] Mars RB, Neubert F-X, Verhagen L, Sallet J, Miller KL, Dunbar RIM, Barton RA 2014 Primate comparative neuroscience using magnetic resonance imaging: promises and challenges. Front. Neurosci. 8, 298 (10.3389/fnins.2014.00298) 25339857 PMC4186285

[150] Mantini D, Hasson U, Betti V, Perrucci MG, Romani GL, Corbetta M, Orban GA, Vanduffel W 2012 Interspecies activity correlations reveal functional correspondence between monkey and human brain areas. Nat. Methods 9, 277–282. (10.1038/nmeth.1868) 22306809 PMC3438906

[151] Mandino F et al. 2020 Animal functional magnetic resonance imaging: trends and path toward standardization. Front. Neuroinform. 13, 78 (10.3389/fninf.2019.00078) 32038217 PMC6987455

[152] Van Essen DC, Dierker DL 2007 Surface-based and probabilistic atlases of primate cerebral cortex. Neuron 56, 209–225. (10.1016/j.neuron.2007.10.015) 17964241

[153] Mars RB, Sotiropoulos SN, Passingham RE, Sallet J, Verhagen L, Khrapitchev AA, Sibson N, Jbabdi S 2018 Whole brain comparative anatomy using connectivity blueprints. eLife 7, e35237 (10.7554/eLife.35237) 29749930 PMC5984034

[154] Hecht EE, Gutman DA, Bradley BA, Preuss TM, Stout D 2015 Virtual dissection and comparative connectivity of the superior longitudinal fasciculus in chimpanzees and humans. NeuroImage 108, 124–137. (10.1016/j.neuroimage.2014.12.039) 25534109 PMC4324003

[155] Eichert N, Verhagen L, Folloni D, Jbabdi S, Khrapitchev AA, Sibson NR, Mantini D, Sallet J, Mars RB 2019 What is special about the human arcuate fasciculus? Lateralization, projections, and expansion. Cortex 118, 107–115. (10.1016/j.cortex.2018.05.005) 29937266 PMC6699597

[156] Ratering D, Baltes C, Nordmeyer-Massner J, Marek D, Rudin M 2008 Performance of a 200-MHz cryogenic RF probe designed for MRI and MRS of the murine brain. Magn. Reson. Med. 59, 1440–1447. (10.1002/mrm.21629) 18421696

[157] Szulc KU, Lerch JP, Nieman BJ, Bartelle BB, Friedel M, Suero-Abreu GA, Watson C, Joyner AL, Turnbull DH 2015 4D MEMRI atlas of neonatal FVB/N mouse brain development. NeuroImage 118, 49–62. (10.1016/j.neuroimage.2015.05.029) 26037053 PMC4554969

[158] Lerch JP, Yiu AP, Martinez-Canabal A, Pekar T, Bohbot VD, Frankland PW, Henkelman RM, Josselyn SA, Sled JG 2011 Maze training in mice induces MRI-detectable brain shape changes specific to the type of learning. NeuroImage 54, 2086–2095. (10.1016/j.neuroimage.2010.09.086) 20932918

[159] Scholz J, Allemang-Grand R, Dazai J, Lerch JP 2015 Environmental enrichment is associated with rapid volumetric brain changes in adult mice. NeuroImage 109, 190–198. (10.1016/j.neuroimage.2015.01.027) 25595504

[160] Blumenfeld-Katzir T, Pasternak O, Dagan M, Assaf Y 2011 Diffusion MRI of structural brain plasticity induced by a learning and memory task. PLoS ONE 6, e20678 (10.1371/journal.pone.0020678) 21701690 PMC3119075

[161] Sampaio-Baptista C et al. 2013 Motor skill learning induces changes in white matter microstructure and myelination. J. Neurosci. 33, 19 499–19 503. (10.1523/JNEUROSCI.3048-13.2013) PMC3858622 24336716

[162] Sampaio-Baptista C et al. 2020 White matter structure and myelin-related gene expression alterations with experience in adult rats. Prog. Neurobiol. 187, 101770 (10.1016/j.pneurobio.2020.101770) 32001310 PMC7086231

[163] Hofstetter S, Tavor I, Moryosef ST, Assaf Y 2013 Short-term learning induces white matter plasticity in the fornix. J. Neurosci. 33, 12 844–12 850. (10.1523/JNEUROSCI.4520-12.2013) PMC6618548 23904619

[164] Jonckers E, Audekerke JV, Visscher GD, der Linden AV, Verhoye M 2011 Functional connectivity fMRI of the rodent brain: comparison of functional connectivity networks in rat and mouse. PLoS ONE 6, e18876 (10.1371/journal.pone.0018876) 21533116 PMC3078931

[165] Lerch JP et al. 2017 Studying neuroanatomy using MRI. Nat. Neurosci. 20, 314–326. (10.1038/nn.4501) 28230838

[166] Kim T, Masamoto K, Fukuda M, Vazquez A, Kim S-G 2010 Frequency-dependent neural activity, CBF, and BOLD fMRI to somatosensory stimuli in isoflurane-anesthetized rats. NeuroImage 52, 224–233. (10.1016/j.neuroimage.2010.03.064) 20350603 PMC2883664

[167] Silva AC, Koretsky AP 2002 Laminar specificity of functional MRI onset times during somatosensory stimulation in rat. Proc. Natl Acad. Sci. USA 99, 15 182–15 187. (10.1073/pnas.222561899) PMC137564 12407177

[168] Jung WB, Shim H-J, Kim S-G 2019 Mouse BOLD fMRI at ultrahigh field detects somatosensory networks including thalamic nuclei. NeuroImage 195, 203–214. (10.1016/j.neuroimage.2019.03.063) 30946950

[169] Dijkhuizen RM, Ren J, Mandeville JB, Wu O, Ozdag FM, Moskowitz MA, Rosen BR, Finklestein SP 2001 Functional magnetic resonance imaging of reorganization in rat brain after stroke. Proc. Natl Acad. Sci. USA 98, 12 766–12 771. (10.1073/pnas.231235598) PMC60128 11606760

[170] Grandjean J, Schroeter A, Batata I, Rudin M 2014 Optimization of anesthesia protocol for resting-state fMRI in mice based on differential effects of anesthetics on functional connectivity patterns. NeuroImage 102, 838–847. (10.1016/j.neuroimage.2014.08.043) 25175535

[171] Grandjean J et al. 2020 Common functional networks in the mouse brain revealed by multi-centre resting-state fMRI analysis. NeuroImage 205, 116278 (10.1016/j.neuroimage.2019.116278) 31614221 PMC7116112

[172] Balsters JH, Zerbi V, Sallet J, Wenderoth N, Mars RB 2019 Primate homologs of mouse cortico-striatal circuits. *bioRxiv* 834481 (10.7554/eLife.53680) PMC7162658 32298231

[173] Boido D, Rungta RL, Osmanski B-F, Roche M, Tsurugizawa T, Bihan DL, Ciobanu L, Charpak S 2019 Mesoscopic and microscopic imaging of sensory responses in the same animal. Nat. Commun. 10, 1–13. (10.1038/s41467-019-09082-4) 30846689 PMC6405955

[174] Chen X et al. 2019 Mapping optogenetically-driven single-vessel fMRI with concurrent neuronal calcium recordings in the rat hippocampus. Nat. Commun. 10, 1–12. (10.1038/s41467-018-07882-8) 31748553 PMC6868210

[175] Schwalm M et al. 2017 Cortex-wide BOLD fMRI activity reflects locally-recorded slow oscillation-associated calcium waves. eLife 6, e27602 (10.7554/eLife.27602) 28914607 PMC5658067

[176] Lahti KM, Ferris CF, Li F, Sotak CH, King JA 1999 Comparison of evoked cortical activity in conscious and propofol-anesthetized rats using functional MRI. Magn. Reson. Med. 41, 412–416. (10.1002/(SICI)1522-2594(199902)41:2<412::AID-MRM28>3.0.CO;2-3) 10080292

[177] Masamoto K, Kanno I 2012 Anesthesia and the quantitative evaluation of neurovascular coupling. J. Cereb. Blood Flow Metab. 32, 1233–1247. (10.1038/jcbfm.2012.50) 22510601 PMC3390804

[178] van Alst TM, Wachsmuth L, Datunashvili M, Albers F, Just N, Budde T, Faber C 2019 Anesthesia differentially modulates neuronal and vascular contributions to the BOLD signal. NeuroImage 195, 89–103. (10.1016/j.neuroimage.2019.03.057) 30930308

[179] Sirmpilatze N, Baudewig J, Boretius S 2019 Temporal stability of fMRI in medetomidine-anesthetized rats. Sci. Rep. 9, 1–13. (10.1038/s41598-019-53144-y) 31723186 PMC6853937

[180] Brydges NM et al. 2013 Imaging conditioned fear circuitry using awake rodent fMRI. PLoS ONE 8, e54197 (10.1371/journal.pone.0054197) 23349824 PMC3551953

[181] Han Z, Chen W, Chen X, Zhang K, Tong C, Zhang X, Li CT, Liang Z 2019 Awake and behaving mouse fMRI during Go/No-Go task. NeuroImage 188, 733–742. (10.1016/j.neuroimage.2019.01.002) 30611875

[182] Beckmann N, Kneuer R, Gremlich H-U, Karmouty-Quintana H, Blé F-X, Müller M 2007 *In vivo* mouse imaging and spectroscopy in drug discovery. NMR Biomed. 20, 154–185. (10.1002/nbm.1153) 17451175

[183] Schambach SJ, Bag S, Schilling L, Groden C, Brockmann MA 2010 Application of micro-CT in small animal imaging. Methods 50, 2–13. (10.1016/j.ymeth.2009.08.007) 19706326

[184] Zanzonico P 2017 Noninvasive imaging for supporting basic research. In Small animal imaging: basics and practical guide (eds Kiessling F, Pichler BJ, Hauff P), pp. 3–32. Cham, Switzerland: Springer International Publishing.

[185] Kagadis GC, Loudos G, Katsanos K, Langer SG, Nikiforidis GC 2010 *In vivo* small animal imaging: current status and future prospects. Med. Phys. 37, 6421–6442. (10.1118/1.3515456) 21302799

[186] Tolman EC 1948 Cognitive maps in rats and men. Psychol. Rev. 55, 189–208. (10.1037/h0061626) 18870876

[187] Dolins FL, Klimowicz C, Kelley J, Menzel CR 2014 Using virtual reality to investigate comparative spatial cognitive abilities in chimpanzees and humans. Am. J. Primatol. 76, 496–513. (10.1002/ajp.22252) 24390812 PMC4710544

[188] Fyhn M, Hafting T, Treves A, Moser M-B, Moser EI 2007 Hippocampal remapping and grid realignment in entorhinal cortex. Nature 446, 190–194. (10.1038/nature05601) 17322902

[189] Kim M, Jeffery KJ, Maguire EA 2017 Multivoxel pattern analysis reveals 3D place information in the human hippocampus. J. Neurosci. 37, 4270–4279. (10.1523/JNEUROSCI.2703-16.2017) 28320847 PMC5413175

[190] Kim M, Maguire EA 2019 Encoding of 3D head direction information in the human brain. Hippocampus. 29, 619–629. (10.1002/hipo.23060) 30561118 PMC6618148

[191] Grieves RM, Jedidi-Ayoub S, Mishchanchuk K, Liu A, Renaudineau S, Jeffery KJ 2020 The place-cell representation of volumetric space in rats. Nat. Commun. 11, 1–13. (10.1038/s41467-020-14611-7) 32034157 PMC7005894

[192] Roberts G et al. 2019 Towards OPM-MEG in a virtual reality environment. NeuroImage 199, 408–417. (10.1016/j.neuroimage.2019.06.010) 31173906 PMC8276767

[193] Possin KL et al. 2016 Cross-species translation of the Morris maze for Alzheimer's disease. J. Clin. Invest. 126, 779–783. (10.1172/JCI78464) 26784542 PMC4731157

[194] Spieker EA, Astur RS, West JT, Griego JA, Rowland LM 2012 Spatial memory deficits in a virtual reality eight-arm radial maze in schizophrenia. Schizophr. Res. 135, 84–89. (10.1016/j.schres.2011.11.014) 22154760 PMC3288352

[195] Haaker J et al. 2019 Making translation work: harmonizing cross-species methodology in the behavioural neuroscience of Pavlovian fear conditioning. Neurosci. Biobehav. Rev. 107, 329–345. (10.1016/j.neubiorev.2019.09.020) 31521698 PMC7822629

[196] Baas JM, Nugent M, Lissek S, Pine DS, Grillon C 2004 Fear conditioning in virtual reality contexts: a new tool for the study of anxiety. Biol. Psychiatry. 55, 1056–1060. (10.1016/j.biopsych.2004.02.024) 15158423

[197] Shemesh Y, Sztainberg Y, Forkosh O, Shlapobersky T, Chen A, Schneidman E 2013 High-order social interactions in groups of mice. eLife 2, e00759 (10.7554/eLife.00759) 24015357 PMC3762363

[198] Freund J, Brandmaier AM, Lewejohann L, Kirste I, Kritzler M, Krüger A, Sachser N, Lindenberger U, Kempermann G 2013 Emergence of individuality in genetically identical mice. Science 340, 756–759. (10.1126/science.1235294) 23661762

[199] Freund J, Brandmaier AM, Lewejohann L, Kirste I, Kritzler M, Krüger A, Sachser N, Lindenberger U, Kempermann G 2015 Association between exploratory activity and social individuality in genetically identical mice living in the same enriched environment. Neuroscience 309, 140–152. (10.1016/j.neuroscience.2015.05.027) 25987202

[200] Kaplan R, Doeller CF, Barnes GR, Litvak V, Düzel E, Bandettini PA, Burgess N 2012 Movement-related theta rhythm in humans: coordinating self-directed hippocampal learning. PLoS Biol. 10, e1001267 (10.1371/journal.pbio.1001267). 22389627 PMC3289589

[201] Vanderwolf CH 1969 Hippocampal electrical activity and voluntary movement in the rat. Electroencephalogr. Clin. Neurophysiol. 26, 407–418. (10.1016/0013-4694(69)90092-3) 4183562

[202] Bush D, Bisby JA, Bird CM, Gollwitzer S, Rodionov R, Diehl B, Mcevoy AW, Walker MC, Burgess N 2017 Human hippocampal theta power indicates movement onset and distance travelled. Proc. Natl Acad. Sci. USA 114, 12 297–12 302. (10.1073/pnas.1708716114) PMC5699050 29078334

[203] Hall SD, Holliday IE, Hillebrand A, Singh KD, Furlong PL, Hadjipapas A, Barnes GR 2005 The missing link: analogous human and primate cortical gamma oscillations. NeuroImage 26, 13–17. (10.1016/j.neuroimage.2005.01.009) 15862200

[204] Kriegeskorte N, Mur M, Bandettini P 2008 Representational similarity analysis—connecting the branches of systems neuroscience. Front. Syst. Neurosci. 2, 4 (10.3389/neuro.06.004.2008) 19104670 PMC2605405

[205] Edelman S 1995 Representation of similarity in three-dimensional object discrimination. Neural Comput. 7, 408–423. (10.1162/neco.1995.7.2.408) 8974734

[206] Edelman S 1998 Representation is representation of similarities. Behav. Brain Sci. 21, 449–467. (10.1017/S0140525X98001253) 10097019

[207] Kriegeskorte N, Mur M, Ruff DA, Kiani R, Bodurka J, Esteky H, Tanaka K, Bandettini PA 2008 Matching categorical object representations in inferior temporal cortex of man and monkey. Neuron 60, 1126–1141. (10.1016/j.neuron.2008.10.043) 19109916 PMC3143574

[208] Anderson J, Barlow HB, Gregory RL, Edelman S, Duvdevani-Bar S 1997 A model of visual recognition and categorization. Phil. Trans. R. Soc. Lond. B 352, 1191–1202. (10.1098/rstb.1997.0102) 9304686 PMC1692007

[209] Haxby JV, Gobbini MI, Furey ML, Ishai A, Schouten JL, Pietrini P 2001 Distributed and overlapping representations of faces and objects in ventral temporal cortex. Science 293, 2425–2430. (10.1126/science.1063736) 11577229

[210] Barron HC et al. 2020 Neuronal computation underlying inferential reasoning in humans and mice. Cell 183, 228–243. (10.1016/j.cell.2020.08.035) 32946810 PMC7116148

[211] Dubois J, de Berker AO, Tsao DY 2015 Single-unit recordings in the macaque face patch system reveal limitations of fMRI MVPA. J. Neurosci. 35, 2791–2802. (10.1523/JNEUROSCI.4037-14.2015) 25673866 PMC4323541

[212] Reardon PK et al. 2018 Normative brain size variation and brain shape diversity in humans. Science 360, 1222–1227. (10.1126/science.aar2578) 29853553 PMC7485526

[213] Pavlov IP 1928 Lectures on conditioned reflexes: twenty-five years of objective study of the higher nervous activity (behaviour) of animals (ed. WH Gantt) New York, NY: Liverwright Publishing Corporation.

[214] Dickinson A 1994 Instrumental conditioning. In *Animal learning and cognition* (ed NJ Mackintosh), pp. 45–79. San Diego, CA: Academic.

[215] Sutton RS, Barto AG 1990 Time-derivative models of Pavlovian reinforcement. In Learning and computational neuroscience: foundations of adaptive networks (eds M Gabriel, J Moore), pp. 497–537. Cambridge, MA: The MIT Press.

[216] Rescorla RA, Wagner AR 1972 A theory of Pavlovian conditioning: variations in the effectiveness of reinforcement and non-reinforcement. In: Classical conditioning II: current research and theory, vol. 2 (eds AH Black, WF Prokasy), pp. 64–99. Norwalk, CT: Appleton-Century-Crofts.

[217] Montague PR, Dayan P, Sejnowski TJ 1996 A framework for mesencephalic dopamine systems based on predictive Hebbian learning. J. Neurosci. 16, 1936–1947. (10.1523/JNEUROSCI.16-05-01936.1996) 8774460 PMC6578666

[218] Schultz W, Dayan P, Montague PR 1997 A neural substrate of prediction and reward. Science 275, 1593–1599. (10.1126/science.275.5306.1593) 9054347

[219] O'Doherty JP, Dayan P, Friston K, Critchley H, Dolan RJ 2003 Temporal difference models and reward-related learning in the human brain. Neuron 38, 329–337. (10.1016/S0896-6273(03)00169-7) 12718865

[220] Rolls ET 2013 The mechanisms for pattern completion and pattern separation in the hippocampus. Front. Syst. Neurosci. 7 (10.3389/fnsys.2013.00074) PMC3812781 24198767

[221] Horner AJ, Bisby JA, Bush D, Lin W-J, Burgess N 2015 Evidence for holistic episodic recollection via hippocampal pattern completion. Nat. Commun. 6, 7462 (10.1038/ncomms8462) 26136141 PMC4506995

[222] Shadlen MN, Newsome WT 2001 Neural basis of a perceptual decision in the parietal cortex (area LIP) of the Rhesus monkey. J. Neurophysiol. 86, 1916–1936. (10.1152/jn.2001.86.4.1916) 11600651

[223] Wang X-J 2002 Probabilistic decision making by slow reverberation in cortical circuits. Neuron 36, 955–968. (10.1016/S0896-6273(02)01092-9) 12467598

[224] Froemke RC, Merzenich MM, Schreiner CE 2007 A synaptic memory trace for cortical receptive field plasticity. Nature 450, 425–429. (10.1038/nature06289) 18004384

[225] Vogels TP, Sprekeler H, Zenke F, Clopath C, Gerstner W 2011 Inhibitory plasticity balances excitation and inhibition in sensory pathways and memory networks. Science 334, 1569–1573. (10.1126/science.1211095) 22075724

[226] Hunt LT, Kolling N, Soltani A, Woolrich MW, Rushworth MFS, Behrens TEJ 2012 Mechanisms underlying cortical activity during value-guided choice. Nat. Neurosci. 15, 470–476, S1–S3 (10.1038/nn.3017) 22231429 PMC3378494

[227] Kay KN, Naselaris T, Prenger RJ, Gallant JL 2008 Identifying natural images from human brain activity. Nature 452, 352–355. (10.1038/nature06713) 18322462 PMC3556484

[228] Dumoulin SO, Wandell BA 2008 Population receptive field estimates in human visual cortex. NeuroImage 39, 647–660. (10.1016/j.neuroimage.2007.09.034) 17977024 PMC3073038

[229] Khaligh-Razavi S-M, Kriegeskorte N 2014 Deep supervised, but not unsupervised, models may explain IT cortical representation. PLoS Comput. Biol. 10, e1003915 (10.1371/journal.pcbi.1003915) 25375136 PMC4222664

[230] Hall C, Lueshen E, Mošat’ A, Linninger AA 2012 Interspecies scaling in pharmacokinetics: a novel whole-body physiologically based modeling framework to discover drug biodistribution mechanisms *in vivo*. J. Pharm. Sci. 101, 1221–1241. (10.1002/jps.22811) 22105643

[231] Bradshaw-Pierce EL, Eckhardt SG, Gustafson DL 2007 A physiologically based pharmacokinetic model of docetaxel disposition: from mouse to man. Clin. Cancer Res. 13, 2768–2776. (10.1158/1078-0432.CCR-06-2362) 17473210

[232] Thiel C et al. 2015 A systematic evaluation of the use of physiologically based pharmacokinetic modeling for cross-species extrapolation. J. Pharm. Sci. 104, 191–206. (10.1002/jps.24214) 25393841

[233] Paul SM, Mytelka DS, Dunwiddie CT, Persinger CC, Munos BH, Lindborg SR, Schacht AL 2010 How to improve R&D productivity: the pharmaceutical industry's grand challenge. Nat. Rev. Drug Discov. 9, 203–214. (10.1038/nrd3078) 20168317

[234] Friston KJ, Stephan KE, Montague R, Dolan RJ 2014 Computational psychiatry: the brain as a phantastic organ. Lancet Psychiatry 1, 148–158. (10.1016/S2215-0366(14)70275-5) 26360579

[235] Nestler EJ, Hyman SE 2010 Animal models of neuropsychiatric disorders. Nat. Neurosci. 13, 1161–1169. (10.1038/nn.2647) 20877280 PMC3750731

[236] Sena ES, van der Worp HB, Bath PMW, Howells DW, Macleod MR 2010 Publication bias in reports of animal stroke studies leads to major overstatement of efficacy. PLoS Biol. 8, e1000344 (10.1371/journal.pbio.1000344) 20361022 PMC2846857

[237] Mak IW, Evaniew N, Ghert M 2014 Lost in translation: animal models and clinical trials in cancer treatment. Am. J. Transl. Res. 6, 114–118. 24489990 PMC3902221

